# Beyond total BDNF: The proBDNF/mBDNF balance in substance use disorders

**DOI:** 10.1016/j.nbd.2026.107531

**Published:** 2026-07-13

**Authors:** Samantha L. Anema, Marcelo M. Melo, Sarah L. Ferri, Serena B. Gumusoglu, Kim T. Blackwell, Susan Q. Shen

**Affiliations:** a Department of Psychiatry, Carver College of Medicine, University of Iowa, Iowa City, IA, United States of America; b Department of Pediatrics, Carver College of Medicine, University of Iowa, Iowa City, IA, United States of America; c Department of Obstetrics and Gynecology, Carver College of Medicine, University of Iowa, Iowa City, IA, United States of America; d Roy J Carver Department of Biomedical Engineering, College of Engineering, University of Iowa, Iowa City, IA, United States of America

**Keywords:** Brain-derived neurotrophic factor, Substance use disorders, Addiction, proBDNF, mBDNF

## Abstract

Brain-derived neurotrophic factor (BDNF) is a central regulator of synaptic plasticity, neuronal survival, and reward circuitry, all of which have been implicated in substance use disorders (SUDs). While prior research has focused on total BDNF levels, emerging evidence highlights the importance of its two major isoforms, proBDNF and mature BDNF (mBDNF), which exert opposing effects through differential receptor activation. ProBDNF promotes synaptic weakening and neuronal apoptosis, while mBDNF supports synaptic strengthening and neuronal survival. Environmental exposures, such as substances of abuse, may shift the proBDNF/mBDNF balance and thereby influence addiction-related neuroplasticity. This review synthesizes evidence highlighting the need to resolve BDNF isoforms, suggesting that the proBDNF/mBDNF ratio may provide mechanistic insight beyond total BDNF alone in addiction neurobiology. We examine BDNF isoform changes following exposure to alcohol, stimulants, and opioids, integrating clinical and preclinical evidence across stages of addiction (acute exposure, chronic use, withdrawal, and abstinence) and brain regions. Collectively, the evidence supports a model in which the proBDNF/mBDNF balance reflects region-specific neurobiological adaptations across stages of addiction. Future isoform-resolved longitudinal clinical studies, together with mechanistic animal and cellular models, may clarify causal relationships and identify opportunities for therapeutic modulation of BDNF signaling in SUDs.

## 1. Introduction: from total BDNF to isoform balance

Decades after its discovery, BDNF remains one of the most extensively studied regulators of synaptic development, learning, memory, and reward-circuit signaling (Barde et al., 1982). However, the literature regarding alterations in brain-derived neurotrophic factor (BDNF) across brain disorders has remained inconsistent (Wang et al., 2022). Discrepancies in the directionality and magnitude of changes in BDNF levels may reflect context-dependent variation across disease stage, brain region, and molecular state rather than uniform changes in BDNF signaling (Wang et al., 2024).

A major shortcoming of BDNF research has been the reliance on measures of total BDNF protein, which overlooks a fundamental feature of BDNF biology: the existence of two protein isoforms with distinct and often opposing functions. In general, the precursor form, proBDNF, preferentially signals through the p75 neurotrophin receptor (p75NTR, also called NGFR) to promote long-term depression (LTD) and apoptotic pathways (Ma et al., 2022; Plotkin et al., 2014; Sakuragi et al., 2013; Yang et al., 2014), whereas the mature form, mBDNF, binds TrkB receptors to support synaptic development and long-term potentiation (LTP) (Qiu et al., 2025). These opposing signaling programs suggest that synaptic and cellular outcomes may depend not only on total BDNF abundance but also on relative isoform composition. However, most studies of BDNF quantify total protein levels without distinguishing proBDNF from mBDNF, potentially obscuring biologically meaningful and even opposing downstream effects.

As a measure of proBDNF/mBDNF balance, we consider the proBDNF/mBDNF ratio, which may shift due to changes in either or both isoforms. This ratio provides a useful starting point for evaluating isoform balance and facilitates comparison across studies. Multiple factors regulate this ratio, including genetic variation, environmental stressors, neuronal activity, and disease states. Altered isoform states have been reported across a range of brain disorders, including neurodegenerative diseases (e.g., Alzheimer’s disease and Parkinson’s disease) and psychiatric disorders (e.g., depression, bipolar disorder, anxiety disorders, and schizophrenia) (Liberona et al., 2024; Wang et al., 2021). Across disorders, altered proBDNF/mBDNF ratios have been associated with vulnerability and impaired neuroplasticity, with a shift toward mBDNF generally associated with neuroprotection. However, the direction and magnitude of these changes vary across brain regions, experimental models, and clinical contexts, suggesting that changes in BDNF isoform balance may represent a context-dependent yet transdiagnostic phenomenon.

BDNF isoform balance may be particularly relevant to substance use disorders (SUDs), which are characterized by persistent substance use despite adverse consequences. In 2024, 16.8% of individuals aged 12 years or older in the United States met criteria for a past-year SUD (SAMHSA, 2025). Addiction-related neuroadaptations include strengthened cue-reward associations, altered salience processing, and persistent remodeling of reward circuits. These adaptations are fundamentally plasticity-dependent processes. Given the distinct functional roles of proBDNF and mBDNF in neuroplasticity, it is plausible that shifts in their ratio may contribute to SUD pathophysiology, positioning BDNF isoform balance as a potential mechanistic contributor to SUDs. However, a major gap exists with the current understanding of BDNF, as illustrated by a 2024 systematic review of human SUD studies that measured BDNF: among the 50 publications included in that study, only 2 distinguished BDNF isoforms (Mancusi et al., 2024).

In this review, we synthesize emerging evidence linking the proBDNF/mBDNF balance to the neurobiology of substance use disorders (SUDs), including its potential relationships with disease severity and clinical course. We discuss potential molecular mechanisms, integrate findings across experimental paradigms and model systems, consider clinical relevance, and highlight key gaps in knowledge. Here, we focus specifically on addiction-related mechanisms rather than neurodevelopmental effects, which themselves alter BDNF trajectories (Little et al., 2021). Effects of prenatal or indirect exposure (e.g., intergenerational effects) are outside of the scope of this review, although they represent important areas of investigation. Finally, we outline priorities for future isoform-resolved studies of BDNF and discuss how consideration of the proBDNF/mBDNF balance may inform future work on SUD-related neuroplasticity.

## 2. Molecular regulation of the proBDNF/mBDNF balance

**Fig. 1.**
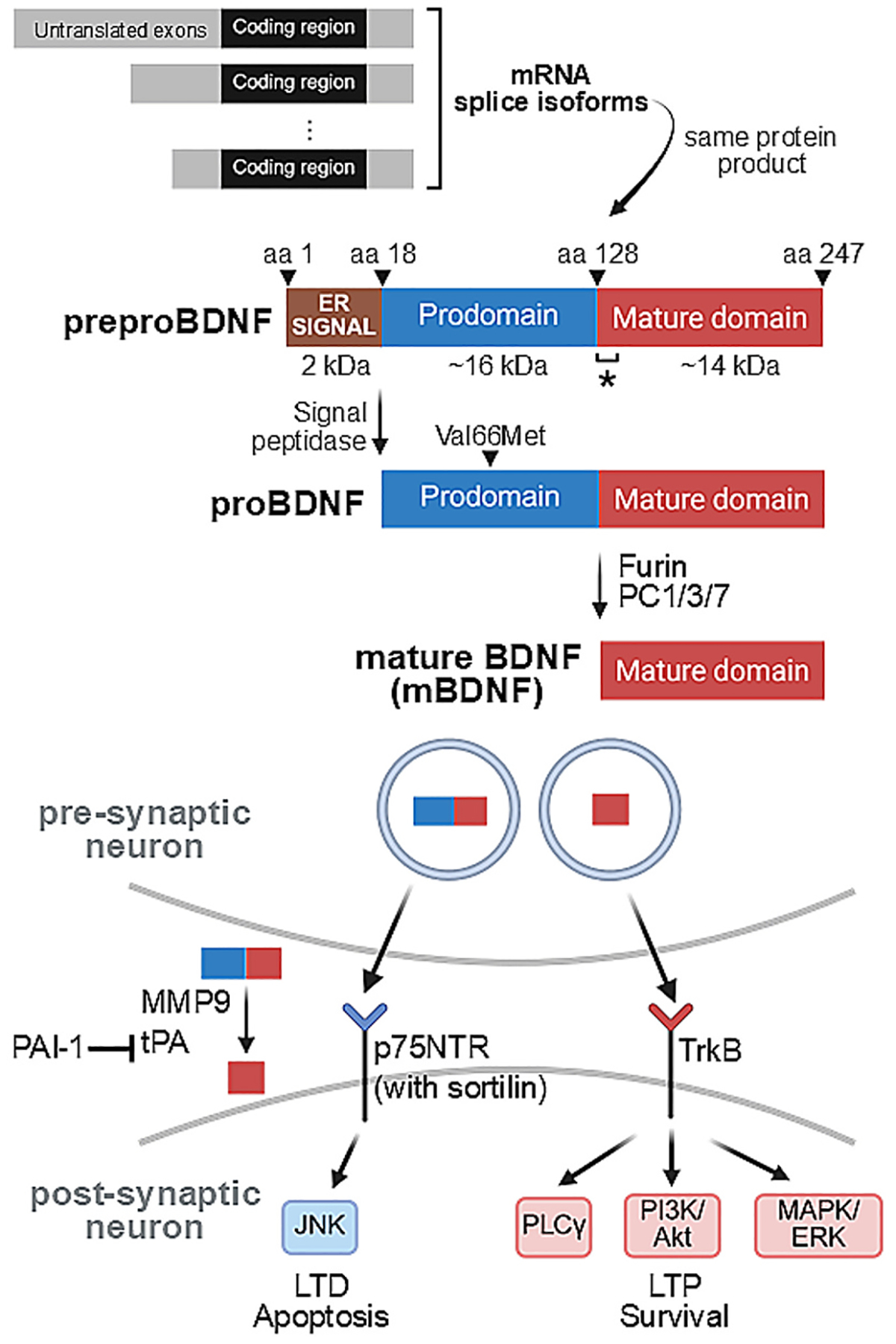
Schematic of BDNF processing. The BDNF gene has numerous splice isoforms that give rise to the same protein product, preproBDNF, which undergoes removal of the ER signaling sequence by signal peptidase. This generates proBDNF, which can be converted to mBDNF via the cleavage of the N-terminal prodomain by intracellular proteases such as Furin and PC1/3/7. Alternatively, proBDNF can be secreted from the cell and remain intact, or proBDNF can be converted to mBDNF by extracellular proteases such as matrix metalloproteinase 9 (MMP9) and the tissue plasminogen activator (tPA)/plasmin system. ProBDNF preferentially binds p75NTR/sortilin, activating downstream pathways such as JNK, promoting long-term depression (LTD) and apoptosis. Mature BDNF preferentially binds TrkB, activating downstream pathways such as PLCγ, promoting long-term potentiation (LTP) and neuronal survival. Note the Val66Met (rs6265) polymorphism located in the prodomain, the inhibition of tPA by PAI-1, and the asterisked region in the mature domain. This asterisk indicates the location of the B-peptide (amino acid sequence HSDPARRC), an epitope that has been used to generate mBDNF-specific antibodies (Nagappan et al., 2009). Figure created in Biorender. Not to scale.

As a foundation for discussing the proBDNF/mBDNF balance in SUDs, we briefly review the established biology of BDNF isoforms and then discuss the potential links with SUDs. Although the *BDNF* locus gives rise to multiple splice isoforms, they differ in untranslated regions and therefore encode the same precursor protein, preproBDNF. PreproBDNF becomes proBDNF upon co-translational removal of the N-terminal endoplasmic reticulum-targeting signaling sequence by signal peptidase (Fig. 1). Functional divergence occurs post-translationally: the prodomain (N-terminus) can be proteolytically cleaved intracellularly or extracellularly to generate mature BDNF (mBDNF). ProBDNF and mBDNF preferentially engage different receptors due to differences in receptor affinity, although there is a lack of precise quantification in the literature, and thereby elicit distinct downstream cellular responses. ProBDNF primarily activates the p75 neurotrophin receptor (p75NTR) and the co-receptor sortilin to promote synaptic pruning, long-term depression (LTD), and apoptosis through pathways including JNK. In contrast, mBDNF predominantly binds TrkB receptors to promote synaptic strengthening, long-term potentiation (LTP), and neuronal survival through pathways including PLCγ (Wang et al., 2022; Wang et al., 2021). Because p75NTR and TrkB are often co-expressed by the same neurons, synaptic and cellular outcomes depend not only on absolute BDNF levels but also on the relative abundance of proBDNF and mBDNF (Yang et al., 2014).

The balance between proBDNF and mBDNF is regulated by cleavage of the proBDNF prodomain (Fig. 1), which in turn is affected by environmental stimuli, making proteolytic processing a key control point linking environmental and genetic factors to downstream neuroplasticity. Intracellular proteases such as Furin and PC1/3/7 convert proBDNF to mBDNF prior to secretion. ProBDNF, mBDNF, or both, can be secreted from the same cell. After secretion of proBDNF, metalloproteinase-9 (MMP9) and the tissue plasminogen activator (tPA)/plasmin system can mediate the extracellular conversion to mBDNF. This is modulated by PAI-1, an inhibitor of tPA (Legutko et al., 2025; Wang et al., 2021). Disruptions in these proteolytic pathways shift the balance toward proBDNF, which is associated with synaptic weakening, maladaptive plasticity, and neurodegeneration (Figiel et al., 2021; Toader et al., 2025).

Importantly, both genetic and non-genetic factors can modulate the proBDNF/mBDNF ratio. Among genetic factors, the BDNF Val66Met (rs6265) polymorphism, located in the prodomain (Fig. 1), is associated with reduced risk for SUDs in some populations, based on candidate gene and genome-wide association studies (Peregud et al., 2026). This polymorphism has been shown to impair BDNF release and could bias the proBDNF/mBDNF ratio in certain contexts (Wang et al., 2022). Beyond genetic factors, neuronal activity also shapes the proBDNF/mBDNF ratio: low-frequency stimulation favors proBDNF release, while high-frequency stimulation induces tPA secretion and increases extracellular mBDNF generation (Nagappan et al., 2009; Yang et al., 2009) and TrkB activation (Legutko et al., 2025). Thus, environmental exposures that alter neuronal activity could dynamically modulate the proBDNF/mBDNF ratio.

Substances of abuse can be viewed as potent environmental perturbations that act on these same regulatory systems, including proteolytic processing, activity-dependent release, and genetic predispositions, potentially altering the proBDNF/mBDNF ratio. For example, variants at the *FURIN* locus are among the strongest genetic associations for opioid use disorders (OUDs) in genome-wide association studies (GWAS’s), suggesting that interindividual differences in the intracellular proBDNF-to-mBDNF conversion may affect OUD risk (Deak et al., 2022). At the same time, variants at the *FURIN* locus are also strongly associated with schizophrenia based on GWAS, and the rs4702 schizophrenia-associated variant in the 3′ untranslated region of *FURIN* is linked to decreased Furin expression and reduced mBDNF production (Hou et al., 2018). Individuals with schizophrenia also have increased risk of OUD and other SUDs (Polimanti et al., 2017). Likewise, a similar pattern is observed in mood and anxiety disorders, which also commonly co-occur with SUDs. These disorders have been associated with elevated proBDNF/mBDNF ratios, thought to be due to impaired proBDNF processing, whereas antidepressant treatment has been linked to restoration of the balance through increased mBDNF levels (Wang et al., 2021). One study found that the proBDNF/mBDNF ratio outperformed either isoform alone in distinguishing unipolar depression (in major depressive disorder) from bipolar depression (in bipolar disorder), supporting the importance of BDNF processing and isoform balance (Zhao et al., 2017). Together, these observations raise the possibility that altered proBDNF-to-mBDNF processing is a mechanistic link underlying shared genetic vulnerability across SUDs and psychiatric disorders.

Notably, different substances engage distinct stress and reward pathways, raising the possibility of substance-specific changes in BDNF signaling. In the following sections, we examine how different classes of substances influence the BDNF isoform balance and incorporate findings regarding interactions with genetic and clinical factors where available. Each section is accompanied by a table to facilitate direct comparison across species and experimental paradigms. These comparisons underscore substance-specific effects within classes of compounds (e.g., different stimulants), brain region-specific responses, and the importance of the exposure timeline.

## 3. Quantifying BDNF isoforms: approaches and limitations

Quantification of BDNF isoforms has primarily relied on two approaches: enzyme-linked immunosorbent assay (ELISA) and Western blot (WB). In general, ELISA is more sensitive than WB and is particularly well suited for measuring secreted protein (e.g., in conditioned media) (Porsch-Ozcurumez et al., 2004). Both approaches depend on antibody-based detection, making epitope selection critical for resolving BDNF isoforms. Antibodies targeting the N-terminus selectively recognize proBDNF, whereas antibodies targeting the C-terminus recognize both proBDNF and mBDNF (Table 1, Fig. 1). Specific detection of mBDNF has been achieved using antibodies directed against the “B-peptide” (depicted by an asterisk in Fig. 1), which is an epitope within the mature domain at the prodomain-mature domain junction that is thought to be sterically inaccessible in intact proBDNF (Nagappan et al., 2009). However, the precise epitopes recognized by commercially available antibodies are often not disclosed.

For ELISA-based approaches, inference of isoform balance requires measurement using at least two of the three antibody classes described above (Table 1). A current limitation of ELISA is that samples must be measured separately for each antibody. Ideally, isoform abundance would be measured simultaneously within the same sample to minimize technical variability, which will require the development of new duplexed assays capable of accurately quantifying both isoforms in the same well (Tighe et al., 2015).

In contrast, Western blot can theoretically resolve both isoforms within a single sample lane using one antibody that recognizes both forms (i.e., targeting the C-terminus). In this case, isoforms are distinguished based on molecular weight, with proBDNF migrating at ~30 kDa and mBDNF at ~15 kDa. However, molecular weight-based quantification has important caveats. First, mBDNF can form dimers with a molecular weight similar to that of monomeric proBDNF (Kolbeck et al., 1994; Radziejewski et al., 1992). This concern is partly mitigated by the use of reducing agents in standard Western blot protocols, which typically disrupt dimers. Second, BDNF undergoes post-translational modifications, including glycosylation of the prodomain, which can produce a broad range of apparent molecular weights (Mowla et al., 2001). Accordingly, molecular weight-based assignment and quantification of BDNF isoforms by Western blot should be interpreted with caution.

Some antibodies and ELISA kits are ambiguous in terms of which isoforms are targeted, simply stating that they measure “BDNF” (without a specifier) and do not provide epitope information (Polacchini et al., 2015). Furthermore, even among BDNF antibodies that are marketed as “specific” to one isoform, there can be cross-reactivity between isoforms. One study found isoform cross-reactivity of 1.3–24.3% across commercially available ELISA kits, as assessed with recombinant proteins (Olivas-Martinez et al., 2025).

To further highlight the need for greater clarity in BDNF research, across studies, there is ambiguity in the use of the term “BDNF”, which may refer either to total BDNF or only mBDNF. Given the above caveats, in the summary tables (Tables 2–4), we list the catalog numbers of antibodies/ELISA kits and use specifiers for BDNF if provided in the publication and/or manufacturer website. For studies reporting changes in both isoforms (or one isoform and total BDNF) but lacking sufficient information to infer relative changes, the inferred ratio is designated as “unclear” in the summary tables (Tables 2–4).

## 4. Selection of biospecimen types for BDNF quantification: considerations and caveats

Another important consideration in BDNF research is the choice of biospecimen. In this section, the discussion is limited to total BDNF, as there is a lack of BDNF isoform-resolved studies that directly compare biospecimen types. BDNF can be measured in primary biofluids, including whole blood, plasma, serum, and cerebrospinal fluid (CSF). Each type of sample likely captures distinct aspects of BDNF biology. The degree to which peripheral total BDNF reflects total brain BDNF also remains incompletely understood, with studies reporting varying degrees of concordance (e.g., (Gejl et al., 2019; Klein et al., 2011; Tikhonova et al., 2022; Yoshimura et al., 2010)).

Several biological factors should be considered when interpreting peripheral BDNF measurements. First, BDNF can cross the blood–brain barrier (BBB), suggesting that peripheral levels could correlate with central BDNF levels. At the same time, disease states and exposures that alter BBB integrity could influence the extent to which BDNF enters the circulation (Pan et al., 1998). Second, peripheral BDNF is not solely derived from the central nervous system. Outside the brain, platelets are the major reservoir of BDNF, storing >90% of blood BDNF, with a portion stored in intracellular α-granules that can be released upon platelet activation. Consequently, peripheral BDNF measurements may reflect a combination of central BDNF signaling and peripheral processes, including platelet abundance and function. This consideration is particularly relevant in SUDs, where platelet biology itself may be altered. For example, chronic alcohol use disorder has been associated with reduced platelet count and impaired platelet function (Mikhailidis et al., 1986). Thus, changes in peripheral BDNF in SUDs may reflect both neurobiological and systemic effects of substance exposure.

In human SUD studies, serum and plasma are the most common biospecimens because of their accessibility (Ornell et al., 2018). However, the choice between serum and plasma has important implications for interpretation of BDNF levels (Serra-Millas, 2016). Serum is collected after allowing blood to clot, a process that activates platelets and induces release of up to 30–40% of platelet BDNF stores. In contrast, plasma is collected in the presence of anticoagulants (e.g., EDTA), minimizing platelet activation and may therefore more closely reflect circulating total BDNF levels. As a result, serum BDNF concentrations are typically 100–200-fold higher than plasma concentrations. Measurements from both sample types are also influenced by pre-analytic variables including storage duration, centrifugation conditions, and platelet contamination. These factors suggest that serum and plasma should not be viewed as interchangeable measures, but rather as reflecting distinct pools of BDNF (Gejl et al., 2019).

Despite these complexities, peripheral BDNF measurements remain valuable due to their potential to capture biologically relevant processes implicated in SUDs, including changes in BBB integrity, systemic inflammation, and platelet function. These considerations emphasize the importance of interpreting peripheral BDNF within the context of biospecimen type and study design. Beyond uncertainty regarding total BDNF, the relationship between proBDNF/mBDNF ratios across biospecimen types remains essentially uncharacterized. Therefore, whether peripheral BDNF isoform balance reflects central BDNF isoform balance remains an important unresolved question. Similar considerations apply to BDNF signaling components: for instance, p75NTR can be shed from the cell membrane and detected in the circulation, but the shedding is a regulated cleavage process, and circulating levels do not necessarily reflect receptor abundance in the brain (Gil et al., 2007) (Weskamp et al., 2004).

## 5. Selection of studies for this review

**Fig. 2.**
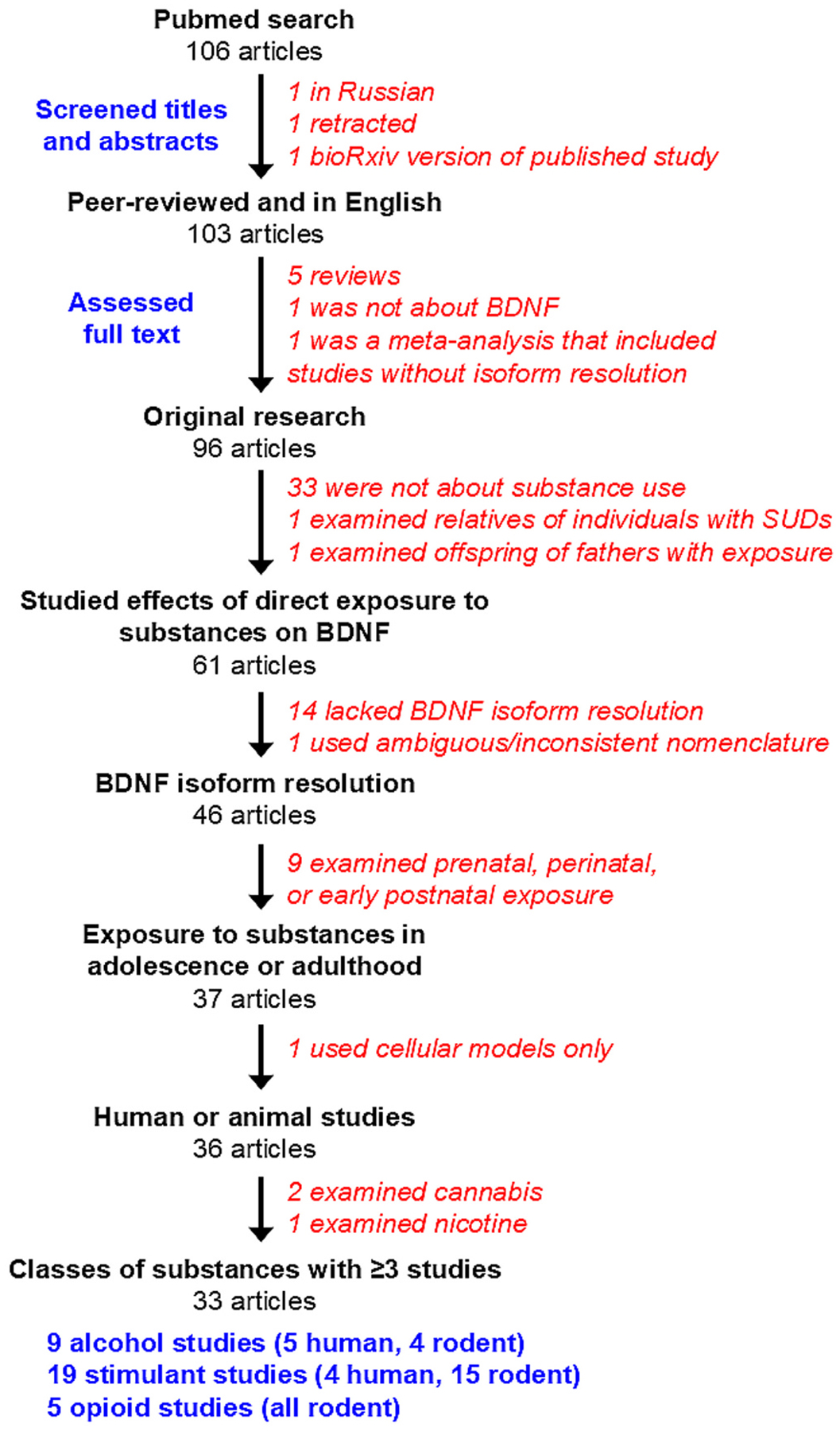
Flow diagram of study selection process. See Section 5 for a detailed description.

To identify research articles for inclusion in this review, we conducted a Pubmed search on May 30, 2026 for “(“BDNF precursor“ OR proBDNF OR pro-BDNF OR “mature BDNF“ OR mBDNF) AND (addiction OR “substance use“ OR alcohol OR ethanol OR stimulant OR methylphenidate OR amphetamine OR cocaine OR methamphetamine OR opioid OR opiate OR nicotine OR cannabis) NOT (caffeine)”. Caffeine was excluded as it is not generally considered a primary substance of abuse. This retrieved 106 articles spanning 2006 to the search date. We required studies to be in English and peer-reviewed, which filtered out 3 articles based on title and abstract. For the remaining 103 articles, we assessed the full text. We required studies to be original research articles in which subjects were directly exposed to substances of abuse. We also required BDNF protein level measurements with isoform resolution enabling assessment of the proBDNF/mBDNF ratio. Hence, 14 studies that did not measure BDNF, measured only total BDNF, or measured one BDNF isoform without measuring total BDNF, were excluded. Additionally, one study was excluded due to within-study inconsistencies (ambiguous BDNF nomenclature, and discrepancy between figures and results description) (Miguez et al., 2020). For reasons described above, we excluded studies of prenatal or perinatal exposure or indirect exposure (e.g., intergenerational effects). Lastly, among the remaining studies, we excluded the only eligible study conducted exclusively in cellular models. We also excluded cannabis and nicotine because the available evidence was too limited, with only two and one eligible studies, respectively. This led to a final list of 33 original research studies (Fig. 2).

In Tables 2–4, we summarize the 33 studies and organize by substance, species, chronicity/stage, and brain region. In the rodent literature, “acute” exposure typically refers to a single dose, “chronic” exposure refers to repeated administration, and “withdrawal” usually refers to a timepoint 24–72 h after the last exposure. We note that “withdrawal” does not necessarily imply physiological withdrawal symptoms. The term “abstinence” can refer to cessation of any duration, while “relapse” indicates re-exposure after abstinence. Detailed study timelines are provided in the text and/or summary tables.

## 6. Alcohol use disorder (AUD) (Table 2)

### 6.1. Epidemiology and significance of AUD

AUD is one of the most prevalent SUDs worldwide and is a major contributor to global morbidity and mortality. In 2024, 9.7% of individuals 12 or older in the United States had an AUD, making up over half of the SUDs in the United States (SAMHSA, 2025). Along with medical and social consequences, AUD is associated with significant neurobiological and cognitive impairment. Clinically, patients with AUD show impairments in executive function, working memory, and episodic memory, consistent with dysfunction in prefrontal and hippocampal circuits that support learning and memory. Neuroimaging studies have reported hippocampal and cortical volume loss, particularly with prolonged alcohol exposure. Notably, improvements in cognitive function and brain structure have been observed following abstinence, suggesting that at least some of the changes in AUD are reversible (Zahr et al., 2011). Together, these features point toward disturbances in synaptic plasticity and neuronal survival, processes regulated by BDNF signaling.

### 6.2. Human studies of AUD suggest differences between the effects of chronic use and withdrawal on BDNF isoform balance

Alcohol-related BDNF studies have focused on chronic AUD and cessation (whether withdrawal or prolonged abstinence) and have been limited to inpatient populations. These studies suggest that chronic AUD is associated with a proBDNF-dominant state in the periphery. Two studies found that chronic AUD is associated with decreased protein levels of mBDNF, TrkB, and tPA, alongside elevated proBDNF, p75NTR, and plasminogen activator inhibitor-1 (PAI-1, which inhibits tPA) in plasma (Fig. 1) (Liu et al., 2024; Mo et al., 2021). These findings are consistent with increased proBDNF signaling and decreased extracellular conversion of proBDNF to mBDNF (Liu et al., 2024). Furthermore, these changes in plasma were modulated by the amount of daily alcohol consumption, meaning that greater alcohol consumption was associated with a stronger bias toward proBDNF (Mo et al., 2021).

Upon cessation of chronic use, the timeline of abstinence may affect the directionality of change. In hospitalized individuals with chronic AUD, within the first 48 h of hospitalization (i.e., presumably days since the last drink), shifts away from mBDNF have been reported in both serum and plasma (Gelle et al., 2024; Mo et al., 2021). With a longer duration of abstinence, there may be a compensatory shift toward mBDNF signaling. Liu et al. (2024) found that compared to when they were actively consuming alcohol, individuals with 2–4 weeks’ abstinence showed a reversal of the proBDNF-dominant pattern in plasma, as assessed by levels of mBDNF, TrkB, and tPA levels (which increased) and proBDNF, p75NTR, and PAI-1 levels (which decreased) (Liu et al., 2024). These findings are consistent with recovery of proteolytic processing and restoration of the proBDNF/mBDNF ratio after prolonged alcohol cessation.

A similar pattern was seen by Malewska-Kasprzak et al. (2025) in hospitalized patients at the beginning and 1 week into the hospitalization. Over the course of seven days, serum proBDNF levels remained unchanged, while total BDNF increased, suggesting a shift toward mBDNF during this period (Malewska-Kasprzak et al., 2025). Similarly, Gelle et al. (2024) found that compared to their isoform levels during a hospitalization, patients had a shift toward mBDNF two months later, regardless of relapse episodes (Gelle et al., 2024). Thus, periods of cessation were correlated with movement toward restoration of the BDNF isoform balance, even if periods of abstinence were punctuated by relapses. These findings suggest that neurobiological recovery may occur with reductions in substance use and not just complete cessation.

### 6.3. Animal studies of alcohol suggest region- and phase-specific changes in the BDNF isoform balance

Animal studies have found that shifts in the proBDNF/mBDNF ratio as related to alcohol are brain-region dependent. In chronic AUD rodent models, prefrontal cortex (PFC) was found in two studies to have decreased mBDNF, although one found decreased proBDNF and the other found no change (Kipp et al., 2024; Lin et al., 2019). Among the available studies, Lin et al. (2019) and Popova et al. (2020) employed the most directly comparable designs, exposing 8-week-old mice to ethanol for approximately one month (Lin et al., 2019; Popova et al., 2020). Across both studies, mBDNF changes were more consistent than proBDNF changes. Both reported decreased mBDNF in hippocampus and no change in hypothalamic mBDNF, whereas proBDNF findings differed between studies (Lin et al., 2019; Popova et al., 2020).

Additionally, alterations in BDNF isoform balance may vary across stages of addiction in rodent models, with restoration of the balance after abstinence, as in humans, although this based on a single rat study in which rats were given alcohol intermittently (2 days on, 2 days off) for one month. Rats were sacrificed 2 h 24 h, or 3 weeks after the last exposure, and levels of proBDNF and mBDNF were quantified in both the PFC and nucleus basalis magnocellularis (NBM) (Kipp et al., 2024). At 2 h, in the PFC, ethanol-treated rats had higher proBDNF/mBDNF compared to water-treated controls. By contrast, the NBM did not show a difference between the two groups. At 24 h, in the PFC, PFC showed lower proBDNF/mBDNF, whereas NBM showed higher proBDNF/mBDNF, in the exposed rats compared to controls. At 3 weeks, the proBDNF/mBDNF ratio was indistinguishable in both regions compared to controls (Kipp et al., 2024). Thus, in this study, the changes in the proBDNF/mBDNF ratio depended on brain region and timing, and as in human studies, showed a movement toward normalization with abstinence.

### 6.4. Summary of alcohol use disorders and BDNF isoform balance

Human and animal studies suggest that the proBDNF/mBDNF balance in AUD is dynamic rather than fixed, fluctuating with disease stage, severity, and psychiatric comorbidities (Gelle et al., 2024; Malewska-Kasprzak et al., 2025; Seo et al., 2021; Zhou et al., 2018). While discrepancies between studies remain, multiple studies have found that cessation of substance use (withdrawal or abstinence) is associated with a shift toward restoration of the proBDNF/mBDNF balance. These findings support a model in which alcohol exposure perturbs isoform balance, while in withdrawal, compensatory adaptations and individual vulnerability factors modulate the direction and magnitude of these shifts.

## 7. Stimulant use disorder (Table 3)

### 7.1. Epidemiology and significance of stimulant use disorders

Stimulant use disorders represent a significant public health burden, with high relapse rates and substantial psychiatric deficits. In 2024, 1.5% of people aged 12 or older in the United States had a stimulant use disorder (SAMHSA, 2025). Affected individuals show deficits in executive function, attention, and working memory, often during early abstinence, consistent with dysfunction in the mesocorticolimbic system (London et al., 2025). Stimulants such as cocaine and methamphetamine induce both short- and long-term synaptic changes that contribute to tolerance, withdrawal, and behavioral sensitization. Chronic exposure leads to persistent alterations in mesolimbic and prefrontal reward circuitry, leading to sensitization and lasting drug-cue associations. These adaptations involve learning and plasticity, processes regulated by BDNF (London et al., 2025).

### 7.2. Cocaine

#### 7.2.1. Human studies suggest that BDNF isoform balance varies by severity and physiological states in cocaine use disorder

Although human studies of BDNF in cocaine use disorder suggest stage-specific alterations, isoform-resolved studies remain scarce (Miuli et al., 2022a). In a study of 28 individuals with at least 48 h of cocaine withdrawal, both total BDNF and proBDNF levels were increased in serum compared with non-users, whereas the proBDNF/mBDNF ratio was unchanged (Pettorruso et al., 2024). In a different study of 24 individuals with at least 48 h of cocaine withdrawal, lower serum proBDNF/mBDNF ratios were associated with longer cocaine exposure, greater amounts used, and more severe insomnia (Miuli et al., 2022b).

#### 7.2.2. Rodent studies suggest that BDNF isoform balance varies by brain region and chronicity in cocaine use

Preclinical studies similarly support phase-dependent regulation of BDNF signaling across stimulant exposure and abstinence. Across brain regions, stimulant-induced changes show distinct patterns. In the PFC, chronic cocaine exposure in rats was associated with reduced proBDNF and mBDNF protein levels despite increased transcription, suggesting altered post-transcriptional processing or turnover (Fumagalli et al., 2007). In contrast, in the striatum for the same study, chronic cocaine exposure was associated with increased proBDNF, while mBDNF levels remained unchanged, indicating an increase in the proBDNF/mBDNF ratio (Fumagalli et al., 2007). The cerebellum also appears sensitive to BDNF processing, with selective increases in proBDNF and p75NTR in a mouse model of relapse followed by withdrawal, indicating an increase in the proBDNF/mBDNF ratio (Vazquez-Sanroman et al., 2015b).

Periods of abstinence appear to partially reverse these changes. Following 45 days of abstinence after adolescent cocaine exposure, rats showed increased proBDNF, mBDNF, and TrkB in the medial PFC, opposite to the reductions observed during chronic exposure (Giannotti et al., 2014). Similar increases in BDNF-related signaling, including proBDNF, mBDNF, and tPA, were observed in the cerebellar vermis following one month of abstinence with a relapse episode (Vazquez-Sanroman et al., 2015a). In contrast, no differences were observed in hippocampal BDNF isoforms following prolonged abstinence compared to non-treated controls in a long-term abstinence model, suggesting restoration toward baseline (Gil-Rodriguez et al., 2025). Thus, periods of withdrawal and abstinence may facilitate restoration of isoform balance, although effects appear region dependent.

### 7.3. Methamphetamine and amphetamine

#### 7.3.1. Human studies suggest shifts toward restoration of the proBDNF/mBDNF ratio with long-term reductions in methamphetamine use

Human studies on methamphetamine have primarily been conducted during longer periods of cessation of use following hospitalization. Two studies evaluated mBDNF and/or proBDNF after cessation of use, and direct comparison is limited by differences in timelines and biospecimen type. In the first study, serum from 85 individuals with methamphetamine use disorder who were in withdrawal or abstinent (up to 30 days since last use) showed decreases in both mBDNF and proBDNF compared to healthy controls (Cheng et al., 2019). In the second study, plasma from 72 individuals abstinent from methamphetamine for 2 years did not show any correlation between the prior duration of methamphetamine use and the levels of either BDNF isoform (Yang et al., 2020). Cognitive status may modulate these patterns, as among the 85 patients in the first study, the 52 individuals with cognitive impairment (as assessed with the Montreal Cognitive Assessment) showed reduced proBDNF and even further reduced mBDNF (i.e., higher proBDNF/mBDNF ratio) compared to the non-cognitively impaired individuals (Cheng et al., 2019). Notably, the identified human studies were limited to methamphetamine and did not include amphetamine.

#### 7.3.2. Rodent studies suggest stage- and region-specific effects of amphetamine and methamphetamine exposure

Rodent studies of amphetamines and methamphetamine are relatively few and use heterogeneous experimental paradigms, limiting direct comparison across studies. Importantly, although methamphetamine and amphetamine are often presumed to produce similar effects, animal studies suggest that this assumption may not extend to BDNF isoform regulation.

Acute exposure appears to have little effect on hippocampal BDNF isoform balance, although the available studies differ in timing of assessment. In one study, a single dose of methamphetamine caused a decrease in mBDNF without changes in proBDNF in the hippocampus of late-adolescent male rats when tissue was collected immediately after exposure (Garcia-Cabrerizo et al., 2018). In contrast, another study found no changes in either isoform 24 h after a single dose of methamphetamine or amphetamine (Garcia-Cabrerizo and Garcia-Fuster, 2016). Together, these findings suggest that acute psychostimulant exposure may have modest or transient effects on BDNF isoforms, although additional studies are needed.

Following repeated exposure, findings across studies differ by substance and exposure timelines. Two studies examined the hippocampus after chronic exposure in adult male rats. After four days of treatment and 24 h’ withdrawal, methamphetamine increased proBDNF, whereas amphetamine increased both proBDNF and mBDNF (Garcia-Cabrerizo and Garcia-Fuster, 2016). In contrast, for methamphetamine, after seven days of treatment and 48 h’ withdrawal, an increase in mBDNF was seen in the hippocampus (McFadden et al., 2014). These findings highlight the need to distinguish the effects of methamphetamine and amphetamine in future studies of BDNF isoforms.

For cessation following chronic methamphetamine use, two studies showed similar patterns despite using different species, ages of animals, and durations of cessation. In both studies, the hippocampus becomes more proBDNF-dominant. Following one week of methamphetamine cessation, adult male mice exhibited increased hippocampal proBDNF and p75NTR, alongside decreased mBDNF and TrkB, compared to saline-treated controls (Wu et al., 2023). Similarly, after one month of methamphetamine cessation, late-adolescent male rats showed lower hippocampal mBDNF than saline-treated controls (Garcia-Cabrerizo et al., 2018). Together, these findings raise the possibility that after chronic methamphetamine use, abstinence shifts the hippocampus away from mBDNF signaling.

#### 7.3.3. Rodent studies suggest that relapse-like methamphetamine exposure may have limited effects on BDNF isoform balance

Despite the heterogeneity of study designs, a notable pattern emerges across studies modeling methamphetamine relapse: a relapse-like challenge (re-introduction following a period of cessation) does not seem to substantially alter hippocampal BDNF isoform balance. In one study, adolescent rats received binge-like methamphetamine exposure (intraperitoneal injections three times daily for four days) followed by one month of cessation. Animals either underwent sacrifice immediately (i.e., no relapse) or received a single methamphetamine dose (i.e., relapse) prior to tissue collection. Both groups showed an increased hippocampal proBDNF/mBDNF ratio, regardless of relapse status, relative to saline-treated controls (Garcia-Cabrerizo et al., 2018). In another study, adult rats underwent seven days of self-administered methamphetamine followed by 24 h of withdrawal; one group was harvested immediately, whereas another received a relapse-like methamphetamine injection followed by an additional 24 h of withdrawal. Both groups showed a decreased hippocampal proBDNF/mBDNF ratio, regardless of relapse status, relative to controls (McFadden et al., 2014). Although the direction of isoform balance differed across studies, possibly reflecting differences in cessation duration (1 day versus 1 month), the relapse-like challenge itself did not substantially alter the ratio within either study. Together, these findings raise the possibility that duration of cessation may be a more important determinant of hippocampal BDNF isoform balance than a single relapse episode, mirroring observations in humans with AUD in which relapse was not associated with marked changes in circulating BDNF isoforms (Gelle et al., 2024).

For amphetamine relapse, studies are even more difficult to compare because they differed in region and timing of tissue collection despite similar exposure paradigms. Two studies used daily amphetamine exposure for eight days followed by approximately one month of cessation and subsequent re-exposure. However, one examined VTA and NAc immediately after re-exposure and observed increases in both proBDNF and total BDNF in both brain regions (Rosa et al., 2023). The other study examined hippocampus 48 h later after re-exposure (mimicking withdrawal after relapse) and found an increase in mBDNF (Segat et al., 2022). These findings underscore that even when exposure paradigms appear similar, differences in experimental details may substantially influence both the observed results and their interpretation. Other rodent studies highlight that in the context of amphetamine use and relapse, age, exercise, and diet may be important modulators of BDNF isoform balance and could contribute to variability across studies (Rosa et al., 2023; Segat et al., 2022; Trevizol et al., 2015).

### 7.4. Other stimulants

Evidence for other stimulants is limited to rodent studies and suggests that BDNF isoform responses are highly context dependent. Chronic exposure to 3,4-methylenedioxymethamphetamine (MDMA) may have age-dependent effects, for instance reducing both BDNF isoforms in the hippocampus of adolescent rats but only proBDNF in the hippocampus of adult rats (Garcia-Cabrerizo and Garcia-Fuster, 2016). Changes may also be sex-dependent, as seen in multiple brain regions in a study of withdrawal from ethylphenidate, a structural analog of methylphenidate (Robins et al., 2019). Finally, a direct comparison of cocaine with 3,4-Methylenedioxypyrovalerone (MDPV, the psychoactive stimulant in bath salts) found distinct effects on BDNF isoforms in the NAc, highlighting the importance of evaluating individual stimulants rather than generalizing across the class (Duart-Castells et al., 2019).

### 7.5. Summary of stimulant use disorders and BDNF isoform balance

Taken together, these studies suggest that changes in BDNF isoform balance in stimulant use disorders may depend on the specific stimulant, exposure chronicity, brain region, and biological context (e.g., sex, age). Environmental factors, such as diet, sleep disruption, and exercise may further modulate these effects. Regardless, across studies, chronic stimulant exposure and withdrawal/abstinence are associated with distinct and potentially compensatory patterns of isoform regulation.

## 8. Opioid use disorder (Table 4)

### 8.1. Epidemiology and significance of opioid use disorder

In 2024, 7.8 million people 12 or older misused opioids in the United States, and 4.8 million had an opioid use disorder (OUD) (SAMHSA, 2025). Individuals with OUD are at increased risk of a wide range of social and health-related harms, including falls, drownings, incarceration, suicide, and homicide. Overall, opioids are responsible for more deaths each year than motor vehicle accidents (SAMHSA, 2025). Intentional and unintentional opioid overdose, misuse of prescription opioids, and use of non-prescription opioids have fueled one of the most severe public health crises in history. Studies have shown that chronic opioid use induces immune dysregulation, neuronal degeneration, and reduced neurotrophin expression, contributing to neuropsychiatric symptoms. These findings suggest a link between opioid pathophysiology and BDNF imbalance (Kosciuczuk et al., 2022). Below, we evaluate BDNF isoform-resolved studies of opioid exposure, all of which were conducted in rodents and focused on morphine.

### 8.2. Rodent studies suggest that BDNF isoform changes with opioids are stage- and region-specific and may vary by the method of inducing opioid withdrawal

Studies investigating the effects of opioid use on the BDNF isoform balance in vivo have been limited to rodent models. These studies suggest that morphine alters BDNF signaling in a region- and state-dependent manner, although studies have primarily focused on chronic exposure and withdrawal. During morphine exposure, the BDNF isoform balance in frontal reward-related regions generally shifts toward mBDNF (i.e., the proBDNF/mBDNF ratio decreases). In rats, the proBDNF/mBDNF ratio decreased in the frontal cortex during morphine dependence (Ozkula et al., 2023). Similarly, an earlier rat study reported a shift toward mBDNF in the frontal cortex and striatum during both acute and chronic morphine exposure (Bachis et al., 2017).

The effects of withdrawal are less clear and may depend on both the brain region and the method used to induce withdrawal, whether naloxone-precipitated or “spontaneous”, i.e., removal of the substance. In the frontal cortex of rats, one study showed a decreased proBDNF/mBDNF ratio with naloxone-precipitated withdrawal (Ozkula et al., 2023), while another study found increased mBDNF without a change in the ratio with spontaneous withdrawal. This second study also found an increase in the proBDNF/mBDNF ratio in the striatum, a tissue not assessed in the first study (Bachis et al., 2017). In mice, naloxone-precipitated withdrawal after chronic morphine increased mBDNF in the dentate gyrus (DG) and basolateral amygdala (BLA), alongside elevated plasma corticosterone and reduced pERK1/2 signaling (Martinez-Laorden et al., 2020). However, in the hippocampus, spontaneous morphine withdrawal increased proBDNF, p75NTR, and p-JNK without affecting mBDNF/TrkB/pERK/pCREB signaling (Martins et al., 2020). Together, these findings suggest that morphine withdrawal induces region-specific shifts in BDNF isoforms, possibly dependent on the withdrawal mechanism.

Only one study was identified that examined the BDNF isoform balance following prolonged abstinence for opioid exposure. In this study, compared to saline-treated controls, postoperative rats treated with morphine for 7 days followed by 21 days of cessation had decreased mBDNF and decreased TrkB signaling in the hippocampus, although no differences were seen in the amygdala (Muscat et al., 2023). These findings emphasize the need for longer-term studies to clarify whether there is eventual restoration of the proBDNF/mBDNF ratio.

### 8.3. Rodent studies suggest a role for inflammatory responses in opioid-induced changes in BDNF isoform balance

Beyond region- and stage-specific effects, inflammatory and stress-related pathways may contribute to opioid-related changes in BDNF isoform balance. In mice, treatment with an antagonist of the corticotropin-releasing factor receptor 1 (CRF1) blocked naloxone-induced increases in DG and BLA mBDNF and plasma corticosterone (Martinez-Laorden et al., 2020). Other studies had demonstrated that CRF1-null mice did not show the negative affective-like behavior or conditioned place aversion seen in wild-type mice during opiate withdrawal (Contarino and Papaleo, 2005; Garcia-Carmona et al., 2015). Together, these findings suggest a possible link between the hypothalamic-pituitary-adrenal (HPA) axis and BDNF through the CRF1 receptor in the context of opioid withdrawal.

Modulation of receptors involved in inflammation may also influence BDNF isoforms. In postoperative rats treated with morphine for 7 days who were then morphine-free for 21 days, mBDNF levels were decreased in the hippocampus (day 28). However, intrathecal administration of lipopolysaccharide from *Rhodobacter sphaeroides* (LPS-RS, a TLR4 antagonist), regardless of whether before or after morphine treatment, restored hippocampal mBDNF levels and the isoform balance (Muscat et al., 2023). Unlike LPS, which triggers inflammation by binding TLR4, LPS-RS is considered non-toxic and competitively binds TLR4 without activating it, ultimately suppressing the inflammatory cascade (Brauckmann et al., 2016). Intrathecal LPS-RS administration was also successful in preventing both the morphine-induced decrease in TrkB and PLCγ phosphorylation/activation (measured on day 28) and the morphine-induced increase in the pro-inflammatory protein HMGB1 (measured on day 10) in the hippocampus. HMGB1 is a damage-associated molecular pattern molecule (DAMP) known to bind several receptors, including TLR4. Uncontrolled or excessive HMGB1 release has been implicated in increased inflammatory responses and cell death (Yuan et al., 2024). These findings point to a potential role for TLR4 in modulating BDNF balance during opioid exposure in the hippocampus.

### 8.4. Summary of opioid use disorders and BDNF isoform balance

Overall, rodent studies indicate that opioid-related changes in BDNF isoform balance are stage-specific and region-specific, with withdrawal being associated with the most pronounced alterations. Importantly, differences between naloxone-precipitated and spontaneous withdrawal paradigms suggest that the withdrawal method itself may substantially influence observed BDNF isoform patterns. Major limitations of the literature on BDNF isoform balance in OUDs are the absence of human studies and the exclusive focus on morphine. Given that individual stimulants may differentially influence the proBDNF/mBDNF ratio (Section 7), it is plausible that other clinically relevant opioids, such as heroin and fentanyl, may exert distinct effects on BDNF isoform balance due to their differing pharmacological properties (Volkow and Blanco, 2021).

## 9. Conclusions and future directions

Overall, the literature suggests that changes in BDNF isoform balance in SUDs are dynamic rather than uniform, varying across substances, stages of addiction, brain regions, and biological context. Across studies, withdrawal and abstinence were often associated with partial restoration of isoform balance, supporting the idea that changes in proBDNF and mBDNF reflect ongoing neuroplastic adaptations rather than fixed disease states. Together, these findings suggest that the proBDNF/mBDNF ratio may capture dimensions of addiction biology that are not evident from total BDNF measurements alone.

Several areas warrant particular attention in future studies. First, methodological standardization and transparency are needed, with consistent nomenclature being a prerequisite. Human studies rely on peripheral biospecimens, particularly serum and plasma. The relationship between peripheral and central BDNF isoform balance remains unresolved, and the relative utility of serum versus plasma may depend on the specific biological question. Interpretation of BDNF isoform measurements is further complicated by variability in sample processing, differences in antibody specificity, and inconsistent BDNF nomenclature.

Second, future work should more systematically investigate biological factors that may shape BDNF isoform balance. Age and sex remain underexplored variables despite evidence across multiple substance classes that these both influence BDNF biology. Although prenatal and developmental exposure studies were outside the scope of this review, they represent important future directions for understanding how early-life environments shape trajectories of BDNF-dependent vulnerability. It also will be essential to examine the intersection with co-occurring conditions. Psychiatric disorders frequently co-occur with SUDs; about half of all individuals with SUDs have a co-occurring psychiatric disorder (SAMHSA, 2025). Psychiatric disorders are themselves associated with altered BDNF signaling, raising the possibility that psychiatric comorbidity influences both baseline isoform states and responses to substance exposure (Peregud et al., 2026). Medical comorbidities associated with SUDs, including HIV, may likewise alter BDNF processing through mechanisms that extend beyond neuronal signaling (Michael et al., 2020).

Third, longitudinal approaches and mechanistic studies are needed to determine whether changes in the proBDNF/mBDNF ratio are causal drivers of addiction-related phenotypes, compensatory adaptations, or manifestations of co-occurring disease states. Isoform-resolved studies spanning acute exposure, chronic exposure, withdrawal, relapse, and recovery will be particularly informative. Pairing behavioral studies with region-specific molecular analyses and experimental perturbation of BDNF processing and receptor signaling may help establish causality and identify therapeutic opportunities. Beyond BDNF isoform levels themselves, future studies in animal and cellular models should incorporate upstream regulators of proBDNF processing (e.g., Furin, tPA/plasmin, MMPs), receptor biology (e.g., TrkB and p75NTR), and downstream signaling pathways to better define the mechanistic links between substance exposure and altered neuroplasticity.

Fourth, future studies should expand the range of substances evaluated. Cannabis and nicotine are among the most commonly used substances in adults, yet there is a paucity of BDNF isoform-resolved studies (SAMHSA, 2025) (Fig. 2). For newer psychoactive substances, including synthetic cannabinoids and emerging hallucinogens, such studies are essentially absent (Shafi et al., 2020). Beyond individual substances, there is an urgent need to investigate polysubstance use, which is highly prevalent among individuals with SUDs. For example, a 2026 study of adults with OUD in the United States reported that polysubstance use was more common than isolated OUD, with 30.1% meeting criteria for two SUDs and 32.4% for three or more SUDs (Han et al., 2026). Polysubstance exposure also occurs unintentionally through adulteration of illicitly manufactured drugs with substances like fentanyl and xylazine (Alexander et al., 2022; Lim et al., 2024). However, systematic investigation of polysubstance effects is a formidable challenge due to the sheer number of possible combinations of substances, doses, and temporal patterns of exposure.

Addressing this complexity will necessitate experimental systems that are both scalable and mechanistically informative. Cellular models are well positioned to meet this need: they enable precise control of dose, timing, combinations of exposures, and genetic background, including patient-specific genetics through the use of induced pluripotent stem cells (Wang et al., 2020). Incorporating cell type-resolved and co-culture systems may further clarify the cell-autonomous and non-cell autonomous mechanisms by which neurons, astrocytes, microglia, and other brain cell types regulate the BDNF isoform balance. Collectively, these capabilities position in vitro studies as an important complement to animal and human studies for advancing our understanding of BDNF isoform biology in SUDs.

From a clinical perspective, the proBDNF/mBDNF ratio may represent a candidate biomarker of addiction stage and neuroplastic state, with potential utility for distinguishing active use, withdrawal, abstinence, and recovery trajectories. If validated longitudinally, the ratio could serve as a biomarker for evaluating interventions and monitoring neurobiological recovery during treatment. Furthermore, because BDNF biology is shaped by both environmental exposures and genetic variation, isoform-resolved measurements may eventually support patient stratification and personalized treatment selection. In terms of therapeutics, selectively modulating the balance between proBDNF and mBDNF may represent a more promising—and more rational—approach than broadly targeting total BDNF. Candidate strategies include enhancing proBDNF intracellular processing by targeting Furin or proprotein convertases, increasing extracellular proBDNF cleavage by targeting MMPs or tPA/plasmin, attenuating p75NTR signaling, or selectively promoting TrkB signaling. Indeed, positive allosteric modulators of TrkB have already advanced to clinical trials as a neuroprotective strategy for neurodegenerative disorders (Forsell et al., 2024). However, for SUDs, translational applications are speculative at present and will require sustained research efforts and long-term collaborations between basic scientists and clinicians.

In conclusion, current evidence suggests that the proBDNF/mBDNF ratio may serve as a useful framework for interpreting addiction-related neuroplasticity. By moving beyond total BDNF measurements and adopting isoform-resolved approaches, future studies may identify more biologically meaningful biomarkers, uncover mechanisms underlying maladaptive plasticity, and establish a stronger foundation for precision diagnostics and therapeutic development. Ultimately, integrating BDNF isoform biology into addiction research may create new opportunities to reduce relapse risk, promote recovery, and improve long-term outcomes in SUDs.

## Figures and Tables

**Table 1:** BDNF antibodies and interpretation by ELISA and Western blot.

Location of epitope	BDNF isoform(s) recognized	ELISA interpretation	Western blot interpretation
Prodomain (N-terminus)	ProBDNF	ProBDNF quantification	ProBDNF band (~30 kDa) densitometry
Mature domain (C-terminus)	ProBDNF and mBDNF	Total BDNF quantification	ProBDNF band (~30 kDa) and mBDNF band (~15 kDa) densitometry
Prodomain-mature domain junction, such as B-peptide in the mature domain (Nagappan et al., 2009)	mBDNF	mBDNF quantification	mBDNF band (~15 kDa) densitometry

**Table 2:** Summary of alcohol use disorder-related studies.

Study	Condition	Species & age	Subjects and timeline	Assay	Tissue	BDNF protein isoforms as measured in study	Inferred proBDNF/mBDNF ratio	Downstream signaling effects
Human Studies: Alcohol								
(Liu et al., 2024)	AUD (baseline)	Humans (24–68 years old)	15 males with AUD, 15 male alcohol non-dependents, 15 healthy control males from Han Chinese population. Blood was collected at baseline, 2 weeks of abstinence, and 4 weeks of abstinence.	ELISA for mBDNF (ELK1394) and proBDNF (ELK8417)	Plasma (baseline)	↓mBDNF ↓proBDNF ↓mBDNF/proBDNF (compared to healthy controls)	↑proBDNF/mBDNF ratio (compared to healthy controls)	↑p75NTR ↓TrkB ↓TrkB/p75NTR (compared to healthy controls)
	Alcohol abstinence (2 weeks and 4 weeks)				Plasma (2 and 4 weeks)	↓proBDNF ↑mBDNF ↑mBDNF/proBDNF (at 2 weeks and 4 weeks compared to AUD baseline)	↓proBDNF/mBDNF (compared to AUD baseline)	↓p75NTR ↑TrkB ↑TrkB/p75NTR (at 2 weeks and 4 weeks compared to AUD baseline)
(Mo et al., 2021)	Alcohol withdrawal	Humans (23–60 years old)	59 alcohol dependents (morning after hospitalization) and 37 healthy controls from Han Chinese population (sexes not specified)	ELISA for proBDNF (R&D Systems, DY3175) and mBDNF (B34D10, custom)	Plasma	↑proBDNF ↓mBDNF (compared to healthy controls) Positive correlation between proBDNF and daily alcohol consumption. Negative correlation between mBDNF and daily alcohol consumption.	↑proBDNF/mBDNF (compared to healthy controls)	N/A
(Gelle et al., 2024)	Alcohol withdrawal (M0)	Humans (40–54 years old)	99 individuals with AUD (79 males and 20 females) in France, and data from 40 healthy controls (extracted from group’s previous publication). Blood was collected at time of alcohol withdrawal (no longer than 48 h after hospitalization) (M0) and two months later (M2). Among the AUD individuals, 45 remained abstinent at M2 and 54 relapsed at M2. History of depressive episodes was also tracked.	ELISA for proBDNF (Aviscera Bioscience SK00752–09) and mBDNF (Aviscera Bioscience SK00752–01, referred to as “BDNF” by authors)	Serum (M0)	↓mBDNF No difference in proBDNF ↓mBDNF/proBDNF (compared to healthy controls)	↑proBDNF/mBDNF (compared to healthy controls)	N/A
	Alcohol abstinence (M2) with or without relapse				Serum (M2)	Compared to healthy controls: ↓mBDNF No difference in proBDNF ↓mBDNF/proBDNF M2 compared to M0 in individuals with a history of AUD: ↑mBDNF No difference in proBDNF ↑mBDNF/proBDNF Relapse status did not affect these patterns In individuals with a history of AUD, depression at M2 was associated with: ↓mBDNF ↓proBDNF No difference in ratio (compared to individuals with a history of AUD without depression at M2)	↑proBDNF/mBDNF (compared to healthy controls) ↓proBDNF/mBDNF (compared to M0) No difference based on depression status at M2 among individuals with AUD	
(Malewska-Kasprzak et al., 2025)	Alcohol withdrawal (≥24 h)	Humans (23–69 years old)	47 Polish males in alcohol withdrawal for at least 24 h and 40 healthy controls Measurements taken on the first and seventh days of hospitalization. Psychiatric diagnoses were noted.	ELISA for total BDNF (R&D Systems DY248) and proBDNF (R&D Systems DY3175)	Serum	Alcohol withdrawal: ↑total BDNF ↓proBDNF (at either timepoint compared to healthy controls) Alcohol withdrawal on day 7 compared to day 1: ↑total BDNF ↓proBDNF Alcohol withdrawal with psychiatric diagnosis: ↑proBDNF (at either timepoint compared to alcohol withdrawal without psychiatric diagnosis; total BDNF not shown for this comparison)	↓proBDNF/mBDNF at either timepoint compared to healthy controls ↓proBDNF/mBDNF over seven days of withdrawal	N/A
(Zhou et al., 2018)	Alcohol withdrawal	Humans (25–63 years old)	30 males with alcohol dependence in a detoxification unit in China and 50 controls. All alcohol-dependent individuals received oral oxazepam (30–60 mg) according to severity of withdrawal symptoms. Blood was collected the morning after admission.	Western Blot for lymphocyte proBDNF (custom) ELISA for serum mBDNF (cB34D10, custom)	Lymphocytes	↑proBDNF (mBDNF not measured) Positive correlation between average daily consumption and proBDNF	Unable to assess because proBDNF and mBDNF were measured in different biospecimens	↑p75NTR ↓TrkB Positive correlation between average daily consumption and p75NTR and sortilin Negative correlation between average daily consumption and TrkB
					Serum	↓mBDNF (proBDNF not measured) Negative correlation between average daily consumption and mBDNF		↓TrkB Negative correlation between average daily consumption and TrkB
Rodent Studies: Alcohol								
(Kipp et al., 2024)	Chronic alcohol (1 month)	Rats (juvenile, P25 at start)	Sprague Dawley juvenile male and female rats treated with 5 g/kg of 20% ethanol through intragastric gavage (2 days on/2 days off) or water (control) on postnatal days 25–57. Sacrificed on the day of the last dose (P57, “chronic”), 24 h after last dose (P58, “withdrawal”), or 3 weeks after last dose (P78, “abstinence”). *N* = 7–10 per condition/sex; no sex differences found	Western blot for BDNF (Abcam ab108319; quantified 25 kDa band as proBDNF and 15 kDa as mBDNF)	PFC (chronic)	↓mBDNF No difference in proBDNF (compared to water controls)	↑proBDNF/mBDNF (compared to water controls)	No differences in p75NTR or TrkB (compared to water controls)
					NBM (chronic)	No differences (compared to water controls)	No difference (compared to water controls)	
	Alcohol withdrawal (24 h)				PFC (withdrawal)	↓proBDNF No difference in mBDNF (compared to water controls)	↓proBDNF/mBDNF (compared to water controls)	
					NBM (withdrawal)	↑proBDNF No difference in mBDNF (compared to water controls)	↑proBDNF/mBDNF (compared to water controls)	
	Abstinence (3 weeks)				PFC (abstinence)	No differences (compared to water controls)	No difference (compared to water controls)	
					NBM (abstinence)	No differences (compared to water controls)	No difference (compared to water controls)	
(Lin et al., 2019)	Chronic alcohol (1 month)	Mice (adults, 8 weeks old at start)	C57BL/6 male and female mice supplied with drinking water containing 1% ethanol (*N* = 16) or no ethanol (*N* = 22) for 4.5 weeks. Tissues were collected shortly after	ELISA for proBDNF and mBDNF (custom)	PFC	↓mBDNF ↓proBDNF No difference in ratio (compared to water controls)	No difference (compared to water controls)	N/A
					Hippocampus	↓mBDNF ↓proBDNF No difference in ratio (compared to water controls)	No difference (compared to water controls)	
					Hypothalamus	↓proBDNF No difference in mBDNF ↓proBDNF/mBDNF (compared to water controls)	↓proBDNF/mBDNF (compared to water controls)	
					Pituitary	↓proBDNF No difference in mBDNF No difference in ratio (compared to water controls)	No difference (compared to water controls)	
					Adrenal	↓mBDNF ↓proBDNF No difference in ratio (compared to water controls)	No difference (compared to water controls)	
(Popova et al., 2020)	Chronic alcohol (6 weeks)	Mice (adults, 8 weeks old at start)	C57BL/6 male mice treated with 10% ethanol bottles or water for 6 weeks. Tissues were collected shortly after. *N* ≥ 6 per group	Western Blot for BDNF (Abcam ab108319; quantified 14 kDa band as mBDNF) and proBDNF (Santa Cruz Biotechnology sc-65,513)	Hypothalamus	No differences (compared to water controls)	No difference (compared to water controls)	No differences in TrkB or p75NTR (compared to water controls)
					Frontal cortex	↑proBDNF No difference in mBDNF (compared to water controls)	↑proBDNF/mBDNF (compared to water controls)	
					Hippocampus	↓mBDNF No difference in proBDNF (compared to water controls)	↑proBDNF/mBDNF (compared to water controls)	
					Amygdala	↑proBDNF No difference in mBDNF (compared to water controls)	↑proBDNF/mBDNF (compared to water controls)	
					Midbrain	↑proBDNF No difference in mBDNF (compared to water controls)	↑proBDNF/mBDNF (compared to water controls)	↑p75NTR No difference in TrkB (compared to water controls)
(Seo et al., 2021)	Alcohol withdrawal (2 days)	Rats (adult, 250–300 g)	Wistar male rats i.p. injected with ethanol 2 g/kg or saline daily for 2 weeks. Sacrificed 2 days after last dose. *N* = 4–6 per group	ELISA for proBDNF (Promega BEK-2217), mBDNF (Promega, BEK-2211)	Amygdala	↓BDNF immunoreactive cell count ↓mBDNF No difference in proBDNF (compared to saline controls)	↑proBDNF/mBDNF (compared to saline controls)	↓pTrkB/TrkB No difference in p75NTR (compared to water controls)

AUD, alcohol use disorder. PFC, prefrontal cortex. NBM, nucleus basalis magnocellularis. i.p., intraperitoneal.

**Table 3:** Summary of stimulant use disorder-related studies.

Study	Condition	Species & age	Subjects	Assay	Tissue	BDNF protein isoforms as measured in study	Inferred proBDNF/mBDNF ratio	Downstream signaling effects
Human Studies: Cocaine								
(Pettorruso et al., 2024)	Cocaine use disorder	Humans (18–65 years old)	28 individuals (27 males and 1 female) with cocaine use disorder and 28 controls from Italy abstinent for at least 48 h	ELISA for proBDNF and mBDNF (custom; mBDNF referred to as “BDNF”; noted to not cross-react with proBDNF)	Serum	↑mBDNF ↑proBDNF No difference in ratio	No difference (compared to healthy controls)	N/A
(Miuli et al., 2022a, 2022b)	Cocaine use disorder	Humans (18–65 years old)	24 individuals (23 male and 1 female) with moderate to severe cocaine use disorder from Italy abstinent for at least 48 h. History of insomnia was noted.	ELISA for proBDNF and mBDNF (custom; mBDNF referred to as “BDNF”; noted to not cross-react with proBDNF)	Serum	↓proBDNF/mBDNF correlated with insomnia, longer history of cocaine use, and higher amount of cocaine used	(directly measured)	N/A
Rodent Studies: Cocaine								
(Fumagalli et al., 2007)	Acute cocaine (1 dose)	Rats (adult, 275–300 g)	Sprague-Dawley male rats i.p. injected with a single dose of cocaine (5 mg/kg, “acute”, with tissue harvested 2 or 24 h after treatment) or once a day for 5 days (5 mg/kg, “chronic”, with tissue harvested 2 h after treatment). An additional “withdrawal” group consisted of chronic treatment followed by 3 days of abstinence. Controls were i.p. saline-treated. *N* = 6–13 per group	Western Blot for BDNF (Santa Cruz Biotechnology; quantified 32 kDa band as proBDNF and 14 kDa band as mBDNF)	PFC (acute)	2 h after treatment: No difference in mBDNF No difference in proBDNF (compared to saline controls) 24 h after treatment: ↑mBDNF No difference in proBDNF (compared to saline controls)	2 h after treatment: No difference (compared to saline controls) 24 h after treatment: ↓proBDNF/mBDNF (compared to saline controls)	N/A
	Chronic cocaine (5 days)				PFC (chronic)	↓proBDNF ↓mBDNF (compared to saline controls)	Unclear	
					Striatum (chronic)	↑proBDNF No difference in mBDNF (compared to saline controls)	↑proBDNF/mBDNF (compared to saline controls)	
	Cocaine withdrawal (3 days)				PFC (withdrawal)	↓proBDNF ↓mBDNF (compared to saline controls) Similar levels as chronic	Unclear	
					Striatum (withdrawal)	↑proBDNF No difference in mBDNF (compared to saline controls) Similar levels as chronic	↑proBDNF/mBDNF (compared to saline controls)	
(Vazquez-Sanroman et al., 2015b)	Cocaine abstinence (1 week), relapse (1 dose), and withdrawal (24 h)	Mice (adult, P77 at start)	Balb/c AnNHsd male mice i.p. injected with cocaine (20 mg/kg) or saline every 48 h for 6 times, followed by a 7-day abstinence period, and then a single injection of cocaine (10 mg/kg) or saline. Tissue was collected 24 h after. *N* = 5–6 per group	Western Blot for proBDNF and mBDNF (Santa Cruz Biotechnology, sc-546; quantified 32 kDa band as proBDNF and 17 kDa band as mBDNF)	Cerebellar vermis	↑proBDNF No difference in mBDNF (compared to saline controls)	↑proBDNF/mBDNF (compared to saline controls)	↑p75NTR
(Giannotti et al., 2014)	Cocaine withdrawal (3 days)	Rats. (adolescent, P28 at start)	Sprague-Dawley adolescent male rats s.c. injected with cocaine (20 mg/kg) or saline once a day for 15 days. The “withdrawal” group consisted of chronic treatment followed by 3 days of cessation. The “abstinence” group was abstinent for 48 days. N = 6 per group	Western Blot for proBDNF (GeneTex) and mBDNF (Santa Cruz Biotechnology)	mPFC (withdrawal)	No differences (compared to saline controls)	No difference (compared to saline controls)	N/A
	Cocaine abstinence (48 days)				mPFC (abstinence)	↑proBDNF ↑mBDNF (compared to saline controls)	Unclear	↑TrkB in crude synaptosomal fraction, but not in the whole PFC homogenate (compared to saline controls)
(Vazquez-Sanroman et al., 2015a)	Cocaine abstinence (1 month), relapse (1 dose), and withdrawal (24h)	Mice (adult, 8 weeks old at start)	Balb/c AnNHsd male mice i.p. injected with cocaine (20 mg/kg) or saline every 48 h for 6 doses, followed by a 1-month withdrawal period, and then a single injection of cocaine (10 mg/kg) or saline. Tissue was collected 24 h after. N = 5 per group	Western Blot for proBDNF and mBDNF (Santa Cruz Biotechnology, sc-546; quantified 32 kDa band as proBDNF and 17 kDa band as mBDNF)	Cerebellar vermis	↑proBDNF ↑mBDNF (compared to saline controls)	Unclear	↑p75NTR ↑TrkB
(Gil-Rodriguez et al., 2025)	Cocaine prolonged abstinence (40 days)	Mice (adolescent, P30 at start)	C57BL/6 J adolescent male mice i.p. injected with cocaine (20 mg/kg) or saline for 21 days. Sacrificed 40 days after last treatment. *N* = 14 per group	Western Blot for”BDNF” (Santa Cruz Biotechnology; quantified 14 kDa band as mBDNF) and proBDNF (Santa Cruz Biotechnology)	Mouse hippocampus	No differences (compared to saline controls)	No difference (compared to saline controls)	N/A
(Duart-Castells et al., 2019)	Chronic cocaine (5 days)	Mice (adolescent, 6 weeks old at start)	Swiss CD-1 adolescent male mice treated with saline (route unspecified), MDPV (1.5 mg/kg s.c.), or cocaine (10 or 15 mg/kg i.p.) daily for 5 days. They were then sacrificed the same day (“chronic”, 2 h after last dose), 24 h after the last dose (“withdrawal”), or 10 days after last dose. N = 5–7 per group	ELISA for mBDNF and proBDNF (Biosensis). ProBDNF was only measured for chronic timepoints.	NAc (cocaine, chronic)	No difference in proBDNF or mBDNF (compared to saline controls)	No difference (compared to saline controls)	N/A
	Cocaine withdrawal (24 h)				NAc (cocaine, withdrawal)	No difference in mBDNF (compared to saline controls)	Unclear (proBDNF not measured)	
	Cocaine abstinence (10 days)				NAc (cocaine, abstinence)	No difference in mBDNF (compared to saline controls)	Unclear (proBDNF not measured)	
	Chronic MDPV (5 days)				NAc (MDPV, chronic)	↓mBDNF No difference in proBDNF (compared to saline controls)	↑proBDNF/mBDNF (compared to saline controls)	
	MDPV withdrawal (24 h)				NAc (MDPV, withdrawal)	No difference in mBDNF (compared to saline controls)	Unclear (proBDNF not measured)	
	MDPV abstinence (10 days)				NAc (MDPV, abstinence)	↑mBDNF (compared to chronic; no difference compared to saline controls)	Unclear (proBDNF not measured)	
Human Studies: Methamphetamine								
(Cheng et al., 2019)	Meth early remission (<30 days)	Humans (18+ years old)	85 males from China with meth use disorder (52 with cognitive impairment) in early (<30 days) remission and 86 healthy controls	ELISA for proBDNF (Shanghai Enzyme-linked Biotechnology ml058345) and mBDNF (Shanghai Enzyme-linked Biotechnology ml900214)	Serum	Meth users compared to controls: ↓mBDNF ↓proBDNF Among cognitively impaired meth users: ↓mBDNF ↓proBDNF (compared to non-impaired users) ↓mBDNF/proBDNF (compared to non-impaired users, and also compared to controls)	↑proBDNF/mBDNF ratio in cognitively impaired users (compared to non-impaired users, and compared to controls)	Meth users compared to controls: ↓TrkB ↓MMP9 Among meth users: ↓MMP9 in cognitively impaired users (compared to non-impaired users)
(Yang et al., 2020)	Meth late remission (2 years)	Humans (23–39 years old)	72 males with meth use disorder (47 with depression and 25 without) from China who were abstinent for 2 years. Those with depression were further divided into two groups (12 weeks of 5 days/week exercise, or 12 weeks sedentary)	ELISA for “BDNF” (specificity unclear; Shanghai Enzyme-linked Biotechnology Company) and proBDNF (Shanghai Enzyme-linked Biotechnology Company)	Plasma	No correlation between duration of meth use and BDNF or proBDNF levels At baseline, in depressed individuals: ↓BDNF No difference in proBDNF (compared to non-depressed individuals) After 12 weeks of exercise, in depressed individuals: ↓BDNF No difference in proBDNF (compared to depressed individuals after 12 weeks sedentary)	Note: although it is unclear whether “BDNF” is total BDNF or mBDNF, since proBDNF levels did not differ, the isoform changes can be inferred At baseline: ↑proBDNF/mBDNF in depressed individuals (compared to non-depressed individuals) Depressed individuals after 12 weeks of exercise: ↑proBDNF/mBDNF (compared to depressed individuals with 12 weeks sedentary)	N/A
Rodent studies: methamphetamine, amphetamine, and other stimulants								
(Garcia-Cabrerizo et al., 2018)	Meth single dose (at age 15 weeks)	Rats (late adolescent, 8 weeks old at start)	Sprague-Dawley late adolescent male rats i.p. injected: With saline 3 times daily for 4 days, followed by a single dose of meth (5 mg/kg) 34 days later (“single dose”) or With meth (5 mg/kg) 3 times daily for 4 days, followed by 34 days of cessation, and a single dose of saline (“abstinence”) or With saline 3 times daily for 4 days, followed by a single dose of saline 34 days later (“saline controls”) or With meth (5 mg/kg) 3 times daily for 4 days, followed by 34 days of cessation, followed by a single dose of meth (5 mg/kg) (“relapse”) N = 6–7 per group	Western Blot for proBDNF and mBDNF (Santa Cruz Biotechnology; quantified 32 kDa band as proBDNF and 14 kDa band as mBDNF)	Hippocampus (acute)	↓mBDNF No difference in proBDNF (compared to saline controls, for any meth treatment regimen)	↑proBDNF/mBDNF (compared to saline controls, for any meth treatment regimen)	N/A
	Meth abstinence (1 month)				Hippocampus (abstinence)			
	Meth relapse (1 month abstinence followed by a single dose)				Hippocampus (relapse)			
(Garcia-Cabrerizo and Garcia-Fuster, 2016)	Short-term MDMA (1 day) followed by 24 h withdrawal	Rats (adolescent, P37 at end, and young adult, P58 at end)	Sprague-Dawley adolescent and young adult male rats i.p. injected with saline or MDMA, meth, or amphetamine (5 mg/kg every 2–3 h, 3 times/day) for 1 day (“short-term”) or 4 days (“chronic”). Tissue was harvested 24 h later. N = 5–6 per group	Western blot for BDNF (Santa Cruz Biotechnology, n-20; quantified 32 kDa band as proBDNF and 14 kDa band as mBDNF)	Hippocampus (MDMA, short-term)	No differences (for either age group compared to saline control)	No difference (for either age group compared to saline control)	N/A
	Chronic MDMA (4 days) followed by 24 h withdrawal				Hippocampus (MDMA, chronic)	P37: ↓proBDNF ↓mBDNF P58: ↓proBDNF No difference in mBDNF (compared to saline controls)	P37: Unclear P58: ↓proBDNF/mBDNF (compared to saline controls)	
	Short-term meth (1 day) followed by 24 h withdrawal				Hippocampus (meth, short-term)	No differences (for either age group compared to saline controls)	No difference (for either age group compared to saline controls)	
	Chronic meth (4 days) followed by 24 h withdrawal				Hippocampus (meth, chronic)	P37: No differences P58: ↑proBDNF No difference in mBDNF (compared to saline controls)	P37: No difference P58: ↑proBDNF/mBDNF (compared to saline controls)	
	Short-term amphetamine (1 day) followed by 24 h withdrawal				Hippocampus (amphetamine, short-term)	No differences (for either age group compared to saline control)	No difference (for either age group compared to saline control)	
	Chronic amphetamine (4 days) followed by 24 h withdrawal				Hippocampus (amphetamine, chronic)	P37: No differences P58: ↑proBDNF ↑mBDNF (compared to saline controls)	Unclear	
(McFadden et al., 2014)	Meth withdrawal (48 h)	Rats (adult, 275–300 g)	Sprague-Dawley male rats self-administered meth (0.12 mg/infusion i.v.) or saline for 7 days. Allowed to withdrawal for 24 h, given saline (4 s.c. injections) and then sacrificed 24 h later (“withdrawal”) or Allowed to withdrawal for 24 h and then given one day of meth (4 s.c. injections of 7.5 mg/kg) and then sacrificed 24 h later (“relapse and withdrawal”) N = 6–14 per group	Western Blot for proBDNF and mBDNF (Santa Cruz Biotechnology; quantified 32 kDa band as proBDNF and 14 kDa band as mBDNF)	Hippocampus (withdrawal)	↑mBDNF No difference in proBDNF (compared to saline controls, regardless of relapse status)	↓proBDNF/mBDNF (compared to saline controls, regardless of relapse status)	N/A
	Meth relapse and withdrawal (24 h)				Hippocampus (relapse and withdrawal)			
(Wu et al., 2023)	Chronic meth (1 week) followed by abstinence (1 week)	Mice (adult, 8 weeks old at start)	C57BL/6 male mice i.p. injected with meth (10 mg/kg) or saline daily for 1 week. Tissue was collected after 1 week	Western Blot for proBDNF (Santa Cruz Biotechnology) and mBDNF (Abcam)	Hippocampus	↓mBDNF ↑proBDNF (compared to saline controls)	↑proBDNF/mBDNF (compared to saline controls)	↓TrkB ↑p75NTR (compared to saline controls)
(Rosa et al., 2023)	Chronic amphetamine (8 days) followed by abstinence (5 weeks), followed by relapse (3 days)	Rats (adult, 150–200 g)	Wistar male rats i.p. injected with amphetamine (4 mg/kg) or saline daily for 8 days, followed by 5 weeks of exercise (or sedentary) with or without naloxone (0.3 mg/kg) injection before, followed by i.p. amphetamine or saline for 3 days. *N* = 4 per group	Western Blot for proBDNF (Abcam) and “BDNF” (Abcam; single band shown, presumably mBDNF)	VTA (sedentary)	↑proBDNF ↑mBDNF (compared to saline controls)	Unclear	↓TrkB (compared to saline controls)
					NAc (sedentary)	↑proBDNF ↑mBDNF (compared to saline controls)	Unclear	↑TrkB (compared to saline controls)
					VTA (exercise)	↑proBDNF ↑mBDNF (compared to sedentary)	Unclear	↑TrkB (compared to sedentary)
					NAc (exercise)	↓proBDNF ↓mBDNF (compared to sedentary)	Unclear	↓TrkB (compared to sedentary)
(Segat et al., 2022)	Chronic amphetamine (8 days) followed by abstinence (4 weeks), relapse (3 days), and withdrawal (48 h)	Rats (adult, 100 g)	Wistar male rats i.p. injected with amphetamine (4 mg/kg) or saline daily for 8 days, followed by 4 weeks of sedentary, non-weight-loaded exercise, or weight-loaded exercise, followed by 3 days of amphetamine or saline conditioning Tissue was collected 48 h after. *N* = 8 per group	Western Blot for proBDNF (Santa Cruz Biotechnology) and mBDNF (Abcam)	Hippocampus (sedentary)	↑mBDNF No difference in proBDNF (compared to saline controls)	↓proBDNF/mBDNF (compared to saline controls)	N/A
					Hippocampus (non-weight-loaded exercise)	↑mBDNF ↑proBDNF (compared to sedentary)	Unclear	
					Hippocampus (weight-loaded exercise)	Compared to non-weight loaded: ↓mBDNF ↓proBDNF	Unclear	
(Trevizol et al., 2015)	Chronic amphetamine (14 days)	Rats (adult, P90)	Wistar male rats that were the offspring from two generations of females supplemented with soybean oil, fish oil, or hydrogenated vegetable oil. Pups were treated with the same supplementation as the dams from weaning to P90. They were then i.p. injected with amphetamine (4 mg/kg) or saline daily for 14 days. Tissue was collected approximately one day after the last i.p. injection. N = 8 per group	Western Blot for proBDNF (Abcam, ab72440) and “BDNF” (Abcam, ab72439, recognizes both isoforms; single band was quantified but band size was unspecified)	Cortex	Soybean oil + amphetamine: ↑proBDNF No difference in mBDNF (compared to soybean oil + saline) Fish oil + amphetamine: ↓BDNF ↓proBDNF (compared to fish oil + saline) Hydrogenated vegetable oil + amphetamine: ↓BDNF No difference in proBDNF (compared to hydrogenated vegetable oil + saline)	Soybean oil and hydrogenated vegetable oil: ↑proBDNF/mBDNF with amphetamine compared to saline Fish oil: ratio unclear	N/A
(Mouri et al., 2017)	Chronic MDMA (7 days)	Mice (adult, 8–12 weeks at start)	C57BL/6 J male mice (with either BDNF+/− or BDNF+/+ genotype) i.p. injected with MDMA (10 mg/kg) or saline daily for 7 days. Tissue was collected 2–3 h after. Sample sizes unclear	Western Blot for BDNF (Santa Cruz Biotechnology; quantified 30 kDa band as proBDNF and 14 kDa band as mBDNF)	NAc	↑proBDNF ↑mBDNF (compared to saline controls)	Unclear	N/A
(Robins et al., 2019)	Chronic ethylphenidate (12 days) followed by withdrawal (3 days)	Mice (adolescent, P42 at start)	C57BL/6 adolescent male and female mice i.p. injected with ethylphenidate (15 mg/kg) or saline daily for 12 days (P42-P53). Tissue was collected 3 days after (on P56). N = 6 per group	Western Blot for BDNF (Abcam ab108319; quantified 32 kDa band as proBDNF and 14 kDa band as mBDNF)	PFC	No differences (for either sex, compared to saline controls)	No difference (for either sex, compared to saline controls)	N/A
					Cortex	Males: ↑proBDNF No difference in mBDNF (compared to male saline controls) Females: No differences (compared to female saline controls)	Males: ↑proBDNF/mBDNF (compared to male saline controls) Females: No difference (compared to female saline controls)	
					Cerebellum			
					Hippocampus	Males: No differences (compared to male saline controls) Females: ↑proBDNF ↑mBDNF (compared to female saline controls)	Males: No difference (compared to male saline control) Females: Unclear	

PFC, prefrontal cortex. mPFC, medial prefrontal cortex. NAc, nucleus accumbens. VTA, ventral tegmental area. MDPV, 3,4-Methylenedioxypyrovalerone. Meth, methamphetamine. MDMA, 3,4-methylenedioxymethamphetamine. i.p., intraperitoneal; s.c., subcutaneous; i.v., intravenous.

**Table 4:** Summary of opioid use disorder-related studies.

Study	Condition	Species & age	Subjects	Assay	Tissue	BDNF protein isoforms as measured in study	Inferred proBDNF/mBDNF ratio	Downstream signaling effects
Rodent Studies: Opioids								
(Ozkula et al., 2023)	Chronic morphine (4 days)	Rats (adult, 250–350 g)	Wistar male rats implanted with slow-release s.c. morphine or placebo pellets (75 mg on day 1 and 300 mg on day 3). One group of morphine-treated rats also received i.p. naloxone (3 mg/kg) to induce withdrawal on day 5. N = 4 per group	Western blot for BDNF (Santa Cruz Biotechnologies sc-20,981) and BDNF (Abcam ab108319). Both show separate bands. Quantified 32 kDa bands as proBDNF and 14 kDa bands as mBDNF, but unclear which antibody was used for which band. (Study states that different isoforms were measured by different antibodies)	Frontal cortex (chronic)	↑mBDNF/proBDNF (compared to placebo controls)	↓proBDNF/mBDNF (compared to placebo controls)	N/A
	Naloxone-induced morphine withdrawal				Frontal cortex (withdrawal)	↑mBDNF/proBDNF (compared to chronic)	↓proBDNF/mBDNF (compared to chronic)	
(Bachis et al., 2017)	Acute morphine (single dose)	Rats (adult, 3 months old)	Sprague-Dawley male rats s.c. injected with a single dose of morphine (10 mg/kg, “acute”) or twice a day for 5 days with escalating doses (10–30 mg/kg, “chronic”). The “withdrawal” group consisted of chronic treatment followed by 2.5 days of abstinence. Controls were s.c. saline-treated. N = 4 per group	For isoform ratio in frontal cortex only: Western blot for BDNF (Promega Corp; quantified 34 kDa band as proBDNF and 14 kDa band as mBDNF) For individual isoform levels in frontal cortex and striatum: ELISA for mBDNF (Aviscera Biosciences) and proBDNF (Promega Corp)	Frontal cortex (acute)	↑mBDNF/proBDNF (compared to saline controls) Quantification of individual isoforms not shown	↓proBDNF/mBDNF (compared to saline controls)	N/A
	Chronic morphine (5 days)				Frontal cortex (chronic)	↑mBDNF ↑proBDNF ↑mBDNF/proBDNF (compared to saline controls)	↓proBDNF/mBDNF (compared to saline controls)	N/A
					Striatum (chronic)	↑mBDNF No difference in proBDNF (compared to saline controls)	↓proBDNF/mBDNF (compared to saline controls)	N/A
	Morphine withdrawal (2.5 days)				Frontal cortex (withdrawal)	Compared to saline controls: ↑mBDNF ↑proBDNF No difference in ratio Compared to chronic: ↓mBDNF ↑proBDNF ↓mBDNF/proBDNF	↑proBDNF/mBDNF (compared to chronic) No difference compared to saline controls	N/A
					Striatum (withdrawal)	Compared to saline controls: ↑proBDNF No difference in mBDNF Compared to chronic: ↓mBDNF ↑proBDNF	↑proBDNF/mBDNF (compared to saline controls, and compared to chronic)	N/A
(Martinez-Laorden et al., 2020)	Naloxone-induced morphine withdrawal	Mice (adult, 25–30 g)	Swiss male mice i.p. injected with escalating doses of morphine (10–60 mg/kg) or saline (8 doses over 4 days). Two hours after the final dose of morphine, mice received a single dose of s.c. naloxone to precipitate withdrawal. N = 5–10 per group	Western blot for BDNF (Santa Cruz Biotechnology; quantified 32 kDa band as proBDNF and 14 kDa band as mBDNF)	Dentate gyrus	↑mBDNF No difference in proBDNF (compared to saline controls)	↓proBDNF/mBDNF (compared to saline controls)	↓pERK1/total ERK ↓pERK2/total ERK (compared to saline controls)
					Basolateral amygdala			
(Martins et al., 2020)	Morphine withdrawal (3 days)	Mice (adult, 2 months old)	Swiss male mice s.c. injected with escalating doses of morphine (20–100 mg/kg) or saline (11 doses over 6 days). Tissue was harvested 3 days after the last dose. N = 5 per group	Western blot for proBDNF (Abcam) and mBDNF (Abcam)	Hippocampus	↑proBDNF No difference in mBDNF (compared to saline controls)	↑proBDNF/mBDNF (compared to saline controls)	↑p75NTR and ↑p-JNK/JNK No difference in TrkB or pERK/ERK (compared to saline controls)
(Muscat et al., 2023)	Morphine abstinence (21 days)	Rats (aged adults, 2 years old)	F344xBN F1 aged male rats underwent sham abdominal surgery, followed by i.p. injection of morphine (2 mg/kg) or saline twice a day for 7 days. After 21 days of abstinence, tissue was harvested. N = 6–7 per group See manuscript text regarding LPS-RS (TLR4 antagonist) and HMGB1	Western blot for BDNF (Abcam ab108319; quantified 32 kDa band as proBDNF and 14 kDa band as mBDNF)	Hippocampus harvested on day 28	↓mBDNF and ↓mBDNF/proBDNF (compared to saline controls)	↑proBDNF/mBDNF (compared to saline controls)	↓pTrkB ↓pTrkB/total TrkB ↓pPLCγ ↓pPLCγ/total PLCγ No differences in total TrkB, pERK, pERK/total ERK or total PLCγ (compared to saline controls)
					Amygdala harvested on day 28	No differences	No difference	No differences

i.p., intraperitoneal; s.c., subcutaneous; ICV, intracerebroventricular.

## Data Availability

Tables 2–4 summarize data from published studies. No additional datasets were used.

## References

[R1] Alexander RS, Canver BR, Sue KL, Morford KL., 2022. Xylazine and overdoses: trends, concerns, and recommendations. Am. J. Public Health 112 (8), 1212–1216. 10.2105/AJPH.2022.306881. 35830662 PMC9342814

[R2] Bachis A, Campbell LA, Jenkins K, Wenzel E, Mocchetti I., 2017. Morphine withdrawal increases brain-derived neurotrophic factor precursor. Neurotox. Res. 32 (3), 509–517. 10.1007/s12640-017-9788-8. 28776309 PMC5711538

[R3] Barde YA, Edgar D, Thoenen H., 1982. Purification of a new neurotrophic factor from mammalian brain. EMBO J. 1 (5), 549–553. 10.1002/j.1460-2075.1982.tb01207.x. 7188352 PMC553086

[R4] Brauckmann S, Effenberger-Neidnicht K, de Groot H, Nagel M, Mayer C, Peters J, Hartmann M., 2016. Lipopolysaccharide-induced hemolysis: evidence for direct membrane interactions. Sci. Rep. 6, 35508. 10.1038/srep35508. 27759044 PMC5069489

[R5] Cheng M, Liu Q, Wang Y, Hao Y, Jing P, Jiao S, Ma L, Pan C, Wu Y., 2019. MMP-9-BDNF pathway is implicated in cognitive impairment of male individuals with methamphetamine addiction during early withdrawal. Behav. Brain Res. 366, 29–35. 10.1016/j.bbr.2019.03.020. 30877026

[R6] Contarino A, Papaleo F., 2005. The corticotropin-releasing factor receptor-1 pathway mediates the negative affective states of opiate withdrawal. Proc. Natl. Acad. Sci. USA 102 (51), 18649–18654. 10.1073/pnas.0506999102. 16339307 PMC1317931

[R7] Deak JD, Zhou H, Galimberti M, Levey DF, Wendt FR, Sanchez-Roige S, Hatoum AS, Johnson EC, Nunez YZ, Demontis D, Børglum AD, Rajagopal VM, Jennings MV, Kember RL, Justice AC, Edenberg HJ, Agrawal A, Polimanti R, Kranzler HR, Gelernter J., 2022. Genome-wide association study in individuals of European and African ancestry and multi-trait analysis of opioid use disorder identifies 19 independent genome-wide significant risk loci. Mol. Psychiatry 27 (10), 3970–3979. 10.1038/s41380-022-01709-1. 35879402 PMC9718667

[R8] Duart-Castells L, Lopez-Arnau R, Vizcaino S, Camarasa J, Pubill D, Escubedo E., 2019. 7,8-Dihydroxyflavone blocks the development of behavioral sensitization to MDPV, but not to cocaine: differential role of the BDNF-TrkB pathway. Biochem. Pharmacol. 163, 84–93. 10.1016/j.bcp.2019.02.004. 30738029

[R9] Figiel I, Kruk PK, Zareba-Koziol M, Rybak P, Bijata M, Wlodarczyk J, Dzwonek J., 2021. MMP-9 signaling pathways that engage Rho GTPases in brain plasticity. Cells 10 (1). 10.3390/cells10010166. PMC7830260 33467671

[R10] Forsell P, Parrado Fernandez C, Nilsson B, Sandin J, Nordvall G, Segerdahl M., 2024. Positive allosteric modulators of Trk receptors for the treatment of Alzheimer’s disease. Pharmaceuticals (Basel) 17 (8). 10.3390/ph17080997. PMC11357672 39204102

[R11] Fumagalli F, Di Pasquale L, Caffino L, Racagni G, Riva MA., 2007. Repeated exposure to cocaine differently modulates BDNF mRNA and protein levels in rat striatum and prefrontal cortex. Eur. J. Neurosci. 26 (10), 2756–2763. 10.1111/j.1460-9568.2007.05918.x. 18001273

[R12] Garcia-Cabrerizo R, Garcia-Fuster MJ., 2016. Comparative effects of amphetamine-like psychostimulants on rat hippocampal cell genesis at different developmental ages. Neurotoxicology 56, 29–39. 10.1016/j.neuro.2016.06.014. 27373168

[R13] Garcia-Cabrerizo R, Bis-Humbert C, Garcia-Fuster MJ., 2018. Methamphetamine binge administration during late adolescence induced enduring hippocampal cell damage following prolonged withdrawal in rats. Neurotoxicology 66, 1–9. 10.1016/j.neuro.2018.02.016. 29501631

[R14] Garcia-Carmona JA, Baroja-Mazo A, Milanes MV, Laorden ML., 2015. Sex differences between CRF1 receptor deficient mice following naloxone-precipitated morphine withdrawal in a conditioned place aversion paradigm: implication of HPA axis. PLoS One 10 (4), e0121125. 10.1371/journal.pone.0121125. 25830629 PMC4382215

[R15] Gejl AK, Enevold C, Bugge A, Andersen MS, Nielsen CH, Andersen LB., 2019. Associations between serum and plasma brain-derived neurotrophic factor and influence of storage time and centrifugation strategy. Sci. Rep. 9 (1), 9655. 10.1038/s41598-019-45976-5. 31273250 PMC6609657

[R16] Gelle T, Vinais T, Lacroix A, Plansont B, Nubukpo P, Girard M., 2024. Serum BDNF and pro-BDNF levels in alcohol use disorders according to depression status: an exploratory study of their evolution two months after withdrawal. Heliyon 10 (19), e38940. 10.1016/j.heliyon.2024.e38940. 39430530 PMC11490827

[R17] Giannotti G, Caffino L, Calabrese F, Racagni G, Riva MA, Fumagalli F., 2014. Prolonged abstinence from developmental cocaine exposure dysregulates BDNF and its signaling network in the medial prefrontal cortex of adult rats. Int. J. Neuropsychopharmacol. 17 (4), 625–634. 10.1017/S1461145713001454. 24345425

[R18] Gil C, Cubi R, Aguilera J., 2007. Shedding of the p75NTR neurotrophin receptor is modulated by lipid rafts. FEBS Lett. 581 (9), 1851–1858. 10.1016/j.febslet.2007.03.080. 17433308

[R19] Gil-Rodriguez S, Berdugo-Gomez M, Claros S, Romero-Zerbo SY, Manas-Padilla MC, Gomez-Roldan MDC, Blanco-Calvo E, Garcia-Fernandez M, Santin LJ., 2025. IGF2 modulates behavioral and hippocampal changes induced by chronic cocaine exposure during adolescence in mice. Pharmacol. Biochem. Behav. 257, 174095. 10.1016/j.pbb.2025.174095. 40925452

[R20] Han B, Volkow ND, Jones CM, Dowell D, Baldwin G, Einstein EB, Subramaniam GA, Olsen Y, Blanco C, Compton WM., 2026. Polysubstance use disorders among US adults. Mol. Psychiatry. 10.1038/s41380-026-03618-z. PMC13442028 42056224

[R21] Hou Y, Liang W, Zhang J, Li Q, Ou H, Wang Z, Li S, Huang X, Zhao C., 2018. Schizophrenia-associated rs4702 G allele-specific downregulation of FURIN expression by miR-338–3p reduces BDNF production. Schizophr. Res. 199, 176–180. 10.1016/j.schres.2018.02.040. 29499969

[R22] Kipp BT, Nunes PT, Savage LM., 2024. Dysregulation of neurotrophin expression in prefrontal cortex and nucleus basalis magnocellularis during and after adolescent intermittent ethanol exposure. Alcohol 120, 1–14. 10.1016/j.alcohol.2024.06.001. 38897258 PMC11390331

[R23] Klein AB, Williamson R, Santini MA, Clemmensen C, Ettrup A, Rios M, Knudsen GM, Aznar S., 2011. Blood BDNF concentrations reflect brain-tissue BDNF levels across species. Int. J. Neuropsychopharmacol. 14 (3), 347–353. 10.1017/S1461145710000738. 20604989

[R24] Kolbeck R, Jungbluth S, Barde YA., 1994. Characterisation of neurotrophin dimers and monomers. Eur. J. Biochem. 225 (3), 995–1003. 10.1111/j.1432-1033.1994.0995b.x. 7957235

[R25] Kosciuczuk U, Jakubow P, Czyzewska J, Knapp P, Rynkiewicz-Szczepanska E., 2022. Plasma brain-derived neurotrophic factor and opioid therapy: results of pilot cross-sectional study. Clin. Med. Res. 20 (4), 195–203. 10.3121/cmr.2022.1731. 36581402 PMC9799226

[R26] Legutko D, Bijoch L, Olszak G, Kuzniewska B, Kalita K, Yasuda R, Kaczmarek L, Michaluk P., 2025. BDNF-driven synaptic plasticity requires autocrine matrix metalloproteinase-9 activity. Sci. Adv. 11 (39), eadx2369. 10.1126/sciadv.adx2369. PMC12459465 40991708

[R27] Liberona A, Jones N, Zuniga K, Garrido V, Zelada MI, Silva H, Nieto RR., 2024. Brain-derived neurotrophic factor (BDNF) as a predictor of treatment response in schizophrenia and bipolar disorder: a systematic review. Int. J. Mol. Sci. 25 (20). 10.3390/ijms252011204. PMC11508575 39456983

[R28] Lim TY, Dong H, Stringfellow E, Hasgul Z, Park J, Glos L, Kazemi R, Jalali MS., 2024. Temporal and spatial trends of fentanyl co-occurrence in the illicit drug supply in the United States: a serial cross-sectional analysis. Lancet Reg. Health Am. 39, 100898. 10.1016/j.lana.2024.100898. 39398941 PMC11470258

[R29] Lin LY, Luo SY, Al-Hawwas M, Herselman MF, Zhou XF, Bobrovskaya L., 2019. The long-term effects of ethanol and corticosterone on the mood-related behaviours and the balance between mature BDNF and proBDNF in mice. J. Mol. Neurosci. 69 (1), 60–68. 10.1007/s12031-019-01328-6. 31127538

[R30] Little B, Sud N, Nobile Z, Bhattacharya D., 2021. Teratogenic effects of maternal drug abuse on developing brain and underlying neurotransmitter mechanisms. Neurotoxicology 86, 172–179. 10.1016/j.neuro.2021.08.007. 34391795

[R31] Liu S, Xie X, Zhao D, Jin N, Hu Y, Wang W, Luo X, Li G, Yang Z., 2024. Alcohol use disorder disrupts BDNF maturation via the PAI-1 pathway which could be reversible with abstinence. Sci. Rep. 14 (1), 22150. 10.1038/s41598-024-73347-2. 39333668 PMC11437282

[R32] London ED, Groman SM, Leyton M, de Wit H., 2025. The mesocorticolimbic system in stimulant use disorder. Mol. Psychiatry 30 (11), 5486–5499. 10.1038/s41380-025-03148-0. 40926091 PMC12532603

[R33] Ma X, Vuyyuru H, Munsch T, Endres T, Lessmann V, Meis S., 2022. ProBDNF dependence of LTD and fear extinction learning in the amygdala of adult mice. Cereb. Cortex 32 (7), 1350–1364. 10.1093/cercor/bhab265. 34470044

[R34] Malewska-Kasprzak M, Permoda-Pachuta A, Skibinska M, Malinowska-Kubiak M, Rybakowski F, Dmitrzak-Weglarz M., 2025. Investigation of serum BDNF levels in alcohol withdrawal syndrome with and without other medical co-morbidities. Alcohol 122, 1–9. 10.1016/j.alcohol.2023.12.006. 38237791

[R35] Mancusi G, Miuli A, Santorelli M, Cavallotto C, Susini O, Pernaci G, Vyborova E, Rosa I, d’Onofrio AM, Camardese G, Pettorruso M, Sensi SL, Martinotti G., 2024. Exploring peripheral biomarkers in psychostimulant use: a systematic review on neurotrophins, stress-related hormones, oxidative stress molecules and genetic factors. Behav. Brain Res. 469, 115046. 10.1016/j.bbr.2024.115046. 38761859

[R36] Martinez-Laorden E, Navarro-Zaragoza J, Milanes MV, Laorden ML, Almela P., 2020. Conditioned aversive memory associated with morphine withdrawal increases brain-derived neurotrophic factor in dentate gyrus and basolateral amygdala. Addict. Biol. 25 (4), e12792. 10.1111/adb.12792. 31282111

[R37] Martins CC, Rosa SG, Recchi AMS, Nogueira CW, Zeni G., 2020. M-trifluoromethyl-diphenyl diselenide (m-CF(3)-PhSe)(2) modulates the hippocampal neurotoxic adaptations and abolishes a depressive-like phenotype in a short-term morphine withdrawal in mice. Prog. Neuro-Psychopharmacol. Biol. Psychiatry 98, 109803. 10.1016/j.pnpbp.2019.109803. 31689445

[R38] McFadden LM, Vieira-Brock PL, Hanson GR, Fleckenstein AE., 2014. Methamphetamine self-administration attenuates hippocampal serotonergic deficits: role of brain-derived neurotrophic factor. Int. J. Neuropsychopharmacol. 17 (8), 1315–1320. 10.1017/S1461145714000327. 24650575 PMC4074226

[R39] Michael H, Mpofana T, Ramlall S, Oosthuizen F., 2020. The role of brain derived neurotrophic factor in HIV-associated neurocognitive disorder: from the bench-top to the bedside. Neuropsychiatr. Dis. Treat. 16, 355–367. 10.2147/NDT.S232836. 32099373 PMC6999762

[R40] Miguez MJ, Bueno D, Espinoza L, Chan W, Perez C., 2020. Among adolescents, BDNF and pro-BDNF lasting changes with alcohol use are stage specific. Neural Plast. 2020, 3937627. 10.1155/2020/3937627. 32399021 PMC7204334

[R41] Mikhailidis DP, Jenkins WJ, Barradas MA, Jeremy JY, Dandona P., 1986. Platelet function defects in chronic alcoholism. Br. Med. J. (Clin. Res. Ed.) 293 (6549), 715–718. 10.1136/bmj.293.6549.715. PMC1341444 3094624

[R42] Miuli A, d’Andrea G, Pettorruso M, Mancusi G, Mosca A, Di Carlo F, Martinotti G, di Giannantonio M., 2022a. From a cycle to a period: the potential role of BDNF as plasticity and phase-specific biomarker in cocaine use disorder. Curr. Neuropharmacol. 20 (11), 2024–2028. 10.2174/1570159X20666220114152052. 35034597 PMC9886838

[R43] Miuli A, Mancusi G, Pettorruso M, Di Carlo F, Clemente K, Di Meo I, D’Andrea A, Pernaci G, Di Crosta T, d’Andrea G, Bubbico G, Martinotti G, Giannantonio MD., 2022b. Impact of sleep disorders and disease duration on neurotrophins levels in cocaine use disorder. Neurosci. Lett. 786, 136805. 10.1016/j.neulet.2022.136805. 35850320

[R44] Mo M, Fu XY, Zhang XL, Zhang SC, Zhang HQ, Wu L, Li JL, Zhou L., 2021. Association of plasma pro-brain-derived neurotrophic factor (proBDNF)/mature brain-derived neurotrophic factor (mBDNF) levels with BDNF gene Val66Met polymorphism in alcohol dependence. Med. Sci. Monit. 27, e930421. 10.12659/MSM.930421. 34415897 PMC8406813

[R45] Mouri A, Noda Y, Niwa M, Matsumoto Y, Mamiya T, Nitta A, Yamada K, Furukawa S, Iwamura T, Nabeshima T., 2017. The involvement of brain-derived neurotrophic factor in 3,4-methylenedioxymethamphetamine-induced place preference and behavioral sensitization. Behav. Brain Res. 329, 157–165. 10.1016/j.bbr.2017.04.052. 28472632

[R46] Mowla SJ, Farhadi HF, Pareek S, Atwal JK, Morris SJ, Seidah NG, Murphy RA., 2001. Biosynthesis and post-translational processing of the precursor to brain-derived neurotrophic factor. J. Biol. Chem. 276 (16), 12660–12666. 10.1074/jbc.M008104200. 11152678

[R47] Muscat SM, Deems NP, Butler MJ, Scaria EA, Bettes MN, Cleary SP, Bockbrader RH, Maier SF, Barrientos RM., 2023. Selective TLR4 antagonism prevents and reverses morphine-induced persistent postoperative cognitive dysfunction, dysregulation of synaptic elements, and impaired BDNF signaling in aged male rats. J. Neurosci. 43 (1), 155–172. 10.1523/JNEUROSCI.1151-22.2022. 36384680 PMC9838714

[R48] Nagappan G, Zaitsev E, Senatorov VV Jr., Yang J, Hempstead BL, Lu B., 2009. Control of extracellular cleavage of ProBDNF by high frequency neuronal activity. Proc. Natl. Acad. Sci. USA 106 (4), 1267–1272. 10.1073/pnas.0807322106. 19147841 PMC2633536

[R49] Olivas-Martinez A, Peinado FM, Perez-Cantero A, Espin-Moreno L, Rodriguez-Carrillo A, Garcia-Esquinas E, Olea N, Mustieles V, Fernandez MF., 2025. A comparison of commercial assays quantifying mature brain-derived neurotrophic factor (mBDNF) and its precursor (pro-BDNF) in human serum. Sci. Rep. 15 (1), 37150. 10.1038/s41598-025-22278-7. 41131273 PMC12550038

[R50] Ornell F, Hansen F, Schuch FB, Pezzini Rebelatto F, Tavares AL, Scherer JN, Valerio AG, Pechansky F, Paim Kessler FH, von Diemen L., 2018. Brain-derived neurotrophic factor in substance use disorders: a systematic review and meta-analysis. Drug Alcohol Depend. 193, 91–103. 10.1016/j.drugalcdep.2018.08.036. 30347311

[R51] Ozkula S, Jafarova Demirkapu M, Yananli HR, Aydin B, Nacar C, Cabadak H., 2023. The effect of acute topiramate administration on morphine withdrawal syndrome and brain-derived neurotrophic factor in central nervous system. Neurol. Res. 45 (8), 730–737. 10.1080/01616412.2023.2203611. 37105528

[R52] Pan W, Banks WA, Fasold MB, Bluth J, Kastin AJ., 1998. Transport of brain-derived neurotrophic factor across the blood-brain barrier. Neuropharmacology 37 (12), 1553–1561. 10.1016/s0028-3908(98)00141-5. 9886678

[R53] Peregud D, Baronets V, Pavlova O, Pavlov K., 2026. BDNF gene polymorphisms and substance use disorders: a systematic review. Rev. Neurosci. 37 (2), 141–188. 10.1515/revneuro-2025-0112. 41188030

[R54] Pettorruso M, Miuli A, Clemente K, Mancusi G, Migliara G, Di Carlo F, Pernaci G, Di Crosta T, Santorelli M, d’Andrea G, De Risio L, Ciavarella M, Baccolini V, Di Meo I, Cataldo I, Sensi SL, Martinotti G., 2024. Enhanced peripheral levels of BDNF and proBDNF: elucidating neurotrophin dynamics in cocaine use disorder. Mol. Psychiatry 29 (3), 760–766. 10.1038/s41380-023-02367-7. 38177347 PMC11153130

[R55] Plotkin JL, Day M, Peterson JD, Xie Z, Kress GJ, Rafalovich I, Kondapalli J, Gertler TS, Flajolet M, Greengard P, Stavarache M, Kaplitt MG, Rosinski J, Chan CS, Surmeier DJ., 2014. Impaired TrkB receptor signaling underlies corticostriatal dysfunction in Huntington’s disease. Neuron 83 (1), 178–188. 10.1016/j.neuron.2014.05.032. 24991961 PMC4131293

[R56] Polacchini A, Metelli G, Francavilla R, Baj G, Florean M, Mascaretti LG, Tongiorgi E., 2015. A method for reproducible measurements of serum BDNF: comparison of the performance of six commercial assays. Sci. Rep. 5, 17989. 10.1038/srep17989. 26656852 PMC4675070

[R57] Polimanti R, Agrawal A, Gelernter J., 2017. Schizophrenia and substance use comorbidity: a genome-wide perspective. Genome Med. 9 (1), 25. 10.1186/s13073-017-0423-3. 28327175 PMC5359801

[R58] Popova NK, Ilchibaeva TV, Antonov EV, Pershina AV, Bazovkina DV, Naumenko VS., 2020. On the interaction between BDNF and serotonin systems: the effects of long-term ethanol consumption in mice. Alcohol 87, 1–15. 10.1016/j.alcohol.2020.04.002. 32330588

[R59] Porsch-Ozcurumez M, Kischel N, Priebe H, Splettstosser W, Finke EJ, Grunow R., 2004. Comparison of enzyme-linked immunosorbent assay, Western blotting, microagglutination, indirect immunofluorescence assay, and flow cytometry for serological diagnosis of tularemia. Clin. Diagn. Lab. Immunol. 11 (6), 1008–1015. 10.1128/CDLI.11.6.1008-1015.2004. 15539498 PMC524736

[R60] Qiu Y, Zhu L, Cai W, Zhu L., 2025. Research progress on BDNF and depression. ACS Chem. Neurosci. 16 (11), 2013–2023. 10.1021/acschemneuro.5c00193. 40359301

[R61] Radziejewski C, Robinson RC, DiStefano PS, Taylor JW., 1992. Dimeric structure and conformational stability of brain-derived neurotrophic factor and neurotrophin-3. Biochemistry 31 (18), 4431–4436. 10.1021/bi00133a007. 1581298

[R62] Robins MT, Blaine AT, Ha JE, Brewster AL, van Rijn RM., 2019. Repeated use of the psychoactive substance ethylphenidate impacts neurochemistry and reward learning in adolescent male and female mice. Front. Neurosci. 13, 124. 10.3389/fnins.2019.00124. 30837836 PMC6389692

[R63] Rosa HZ, Segat HJ, Barcelos RCS, Kr K, R. RD, E. BM., 2023. Physical exercise promotes beneficial changes on neurotrophic factors in mesolimbic brain areas after AMPH relapse: involvement of the endogenous opioid system. Neurotox. Res. 41 (6), 741–751. 10.1007/s12640-023-00675-y. 37904065

[R64] Sakuragi S, Tominaga-Yoshino K, Ogura A., 2013. Involvement of TrkB- and p75 (NTR)-signaling pathways in two contrasting forms of long-lasting synaptic plasticity. Sci. Rep. 3, 3185. 10.1038/srep03185. 24212565 PMC3822391

[R65] SAMHSA, 2025. Key Substance Use and Mental Health Indicators in the United States: Results from the 2024 National Survey on Drug Use and Health (HHS Publication No. PEP25-07-007, NSDUH Series H-60). https://www.samhsa.gov/data/data-we-collect/nsduh-national-survey-drug-use-and-health/national-releases.

[R66] Segat HJ, Martini F, Roversi K, Rosa SG, Muller SG, Rossato DR, Nogueira CW, Burger ME., 2022. Impact of two different types of exercise training on AMPH addiction: role of hippocampal neurotrophins. Physiol. Behav. 251, 113804. 10.1016/j.physbeh.2022.113804. 35398334

[R67] Seo SY, Bang SK, Kang SY, Cho SJ, Choi KH, Ryu YH., 2021. Acupuncture alleviates anxiety and 22-kHz ultrasonic vocalizations in rats subjected to repeated alcohol administration by modulating the brain-derived neurotrophic factor/corticotropin-releasing hormone signaling pathway. Int. J. Mol. Sci. 22 (8). 10.3390/ijms22084037. PMC8070810 33919862

[R68] Serra-Millas M., 2016. Are the changes in the peripheral brain-derived neurotrophic factor levels due to platelet activation? World J. Psychiatry 6 (1), 84–101. 10.5498/wjp.v6.i1.84. 27014600 PMC4804271

[R69] Shafi A, Berry AJ, Sumnall H, Wood DM, Tracy DK., 2020. New psychoactive substances: a review and updates. Ther. Adv. Psychopharmacol. 10, 2045125320967197. 10.1177/2045125320967197. PMC7750892 33414905

[R70] Tighe PJ, Ryder RR, Todd I, Fairclough LC., 2015. ELISA in the multiplex era: potentials and pitfalls. Proteomics Clin. Appl. 9 (3–4), 406–422. 10.1002/prca.201400130. 25644123 PMC6680274

[R71] Tikhonova MA, Zhanaeva SY, Shvaikovskaya AA, Olkov NM, Aftanas LI, Danilenko KV., 2022. Neurospecific molecules measured in periphery in humans: how do they correlate with the brain levels? A systematic review. Int. J. Mol. Sci. 23 (16). 10.3390/ijms23169193. PMC9409387 36012459

[R72] Toader C, Serban M, Munteanu O, Covache-Busuioc RA, Enyedi M, Ciurea AV, Tataru CP., 2025. From synaptic plasticity to neurodegeneration: BDNF as a transformative target in medicine. Int. J. Mol. Sci. 26 (9). 10.3390/ijms26094271. PMC12071950 40362507

[R73] Trevizol F, Roversi K, Dias VT, Roversi K, Barcelos RC, Kuhn FT, Pase CS, Golombieski R, Veit JC, Piccolo J, Pochmann D, Porciuncula LO, Emanuelli T, Rocha JB, Burger ME., 2015. Cross-generational trans fat intake facilitates mania-like behavior: oxidative and molecular markers in brain cortex. Neuroscience 286, 353–363. 10.1016/j.neuroscience.2014.11.059. 25499313

[R74] Vazquez-Sanroman D, Carbo-Gas M, Leto K, Cerezo-Garcia M, Gil-Miravet I, Sanchis-Segura C, Carulli D, Rossi F, Miquel M., 2015a. Cocaine-induced plasticity in the cerebellum of sensitised mice. Psychopharmacology 232 (24), 4455–4467. 10.1007/s00213-015-4072-1. 26482898

[R75] Vazquez-Sanroman D, Leto K, Cerezo-Garcia M, Carbo-Gas M, Sanchis-Segura C, Carulli D, Rossi F, Miquel M., 2015b. The cerebellum on cocaine: plasticity and metaplasticity. Addict. Biol. 20 (5), 941–955. 10.1111/adb.12223. 25619460

[R76] Volkow ND, Blanco C., 2021. The changing opioid crisis: development, challenges and opportunities. Mol. Psychiatry 26 (1), 218–233. 10.1038/s41380-020-0661-4. 32020048 PMC7398847

[R77] Wang M, Zhang L, Gage FH., 2020. Modeling neuropsychiatric disorders using human induced pluripotent stem cells. Protein Cell 11 (1), 45–59. 10.1007/s13238-019-0638-8. 31134525 PMC6949328

[R78] Wang M, Xie Y, Qin D., 2021. Proteolytic cleavage of proBDNF to mBDNF in neuropsychiatric and neurodegenerative diseases. Brain Res. Bull. 166, 172–184. 10.1016/j.brainresbull.2020.11.005. 33202257

[R79] Wang CS, Kavalali ET, Monteggia LM., 2022. BDNF signaling in context: from synaptic regulation to psychiatric disorders. Cell 185 (1), 62–76. 10.1016/j.cell.2021.12.003. 34963057 PMC8741740

[R80] Wang D, Lang ZC, Wei SN, Wang W, Zhang H., 2024. Targeting brain-derived neurotrophic factor in the treatment of neurodegenerative diseases: a review. Neuroprotection 2 (2), 67–78. 10.1002/nep3.43. 41383700 PMC12486910

[R81] Weskamp G, Schlondorff J, Lum L, Becherer JD, Kim TW, Saftig P, Hartmann D, Murphy G, Blobel CP., 2004. Evidence for a critical role of the tumor necrosis factor alpha convertase (TACE) in ectodomain shedding of the p75 neurotrophin receptor (p75NTR). J. Biol. Chem. 279 (6), 4241–4249. 10.1074/jbc.M307974200. 14638693

[R82] Wu Y, Dong Z, Jiang X, Qu L, Zhou W, Sun X, Hou J, Xu H, Cheng M., 2023. Gut microbiota taxon-dependent transformation of microglial M1/M2 phenotypes underlying mechanisms of spatial learning and memory impairment after chronic methamphetamine exposure. Microbiol. Spectrum 11 (3), e0030223. 10.1128/spectrum.00302-23. PMC10269813 37212669

[R83] Yang J, Siao CJ, Nagappan G, Marinic T, Jing D, McGrath K, Chen ZY, Mark W, Tessarollo L, Lee FS, Lu B, Hempstead BL., 2009. Neuronal release of proBDNF. Nat. Neurosci. 12 (2), 113–115. 10.1038/nn.2244. 19136973 PMC2737352

[R84] Yang J, Harte-Hargrove LC, Siao CJ, Marinic T, Clarke R, Ma Q, Jing D, Lafrancois JJ, Bath KG, Mark W, Ballon D, Lee FS, Scharfman HE, Hempstead BL., 2014. proBDNF negatively regulates neuronal remodeling, synaptic transmission, and synaptic plasticity in hippocampus. Cell Rep. 7 (3), 796–806. 10.1016/j.celrep.2014.03.040. 24746813 PMC4118923

[R85] Yang J, Tan J, Zheng L, Lu CX, Hou WQ, Liu Y, Li QF, Li JX, Cheng D, Luo X, Zhang J., 2020. Plasma BDNF and TrkB mRNA in PBMCs are correlated with anti-depressive effects of 12-weeks supervised exercise during protracted methamphetamine abstinence. Front. Mol. Neurosci. 13, 20. 10.3389/fnmol.2020.00020. 32210759 PMC7069447

[R86] Yoshimura R, Sugita-Ikenouchi A, Hori H, Umene-Nakano W, Hayashi K, Katsuki A, Ueda N, Nakamura J., 2010. A close correlation between plasma and serum levels of brain-derived neurotrophic factor (BDNF) in healthy volunteers. Int. J. Psychiatry Clin. Pract. 14 (3), 220–222. 10.3109/13651501003748560. 24917323

[R87] Yuan J, Guo L, Ma J, Zhang H, Xiao M, Li N, Gong H, Yan M., 2024. HMGB1 as an extracellular pro-inflammatory cytokine: implications for drug-induced organic damage. Cell Biol. Toxicol. 40 (1), 55. 10.1007/s10565-024-09893-2. 39008169 PMC11249443

[R88] Zahr NM, Kaufman KL, Harper CG., 2011. Clinical and pathological features of alcohol-related brain damage. Nat. Rev. Neurol. 7 (5), 284–294. 10.1038/nrneurol.2011.42. 21487421 PMC8121189

[R89] Zhao G, Zhang C, Chen J, Su Y, Zhou R, Wang F, Xia W, Huang J, Wang Z, Hu Y, Cao L, Guo X, Yuan C, Wang Y, Yi Z, Lu W, Wu Y, Wu Z, Hong W, Fang Y., 2017. Ratio of mBDNF to proBDNF for differential diagnosis of major depressive disorder and bipolar depression. Mol. Neurobiol. 54 (7), 5573–5582. 10.1007/s12035-016-0098-6. 27613282

[R90] Zhou L, Xiong J, Ruan CS, Ruan Y, Liu D, Bao JJ, Zhou XF., 2018. ProBDNF/p75NTR/sortilin pathway is activated in peripheral blood of patients with alcohol dependence. Transl. Psychiatry 7 (11), 2. 10.1038/s41398-017-0015-4. 29520063 PMC5843592

